# A Type 2 Diabetes Policy Model That Predicts Remaining Life Expectancy, Quality‐Adjusted Life Expectancy and Healthcare Costs for Use in Economic Evaluation, Incorporating Equity Concerns

**DOI:** 10.1111/dom.70657

**Published:** 2026-03-19

**Authors:** Lili Wei, Houra Haghpanahan, Sarah H. Wild, Claudia Geue, Elaine Butterly, Peter McMeekin, Stuart McGurnaghan, Luke A. K. Blackbourn, Robert Lindsay, David McAllister, Jim Lewsey

**Affiliations:** ^1^ School of Health & Wellbeing University of Glasgow Glasgow UK; ^2^ Usher Institute University of Edinburgh Edinburgh UK; ^3^ Institute of Genetics and Cancer University of Edinburgh Edinburgh UK; ^4^ School of Cardiovascular & Metabolic Health University of Glasgow Glasgow UK

**Keywords:** database research, health economics, real‐world evidence, type 2 diabetes

## Abstract

**Aims:**

To illustrate a T2DM policy model incorporating socio‐economic status.

**Materials and Methods:**

Using the Scottish Diabetes Research Network (SDRN) national diabetes cohort, we identified individuals newly diagnosed with T2DM between 1 January 2004 and 31 December 2020 and followed from diagnosis until death or end of follow‐up. Covariates included sex, Scottish Index of Multiple Deprivation (SIMD) quintiles, ethnicity, HbA1c, systolic blood pressure (SBP), eGFR, total cholesterol, high‐density lipoprotein, BMI, smoking status and cardiovascular disease history. Remaining life expectancy was modelled using Gompertz regression, stratified by sex and SIMD. Probabilities of complications were estimated using repeated measure logistic regression, and healthcare costs were modelled using repeated measure linear regression. A parameterised Kaplan–Meier sample‐average estimator was used to obtain life expectancy, quality‐adjusted life years (QALYs) and costs.

**Results:**

We identified 269 759 people newly diagnosed with T2DM. The mean age at diagnosis was 63 years, 56% were male and 24% lived in the most deprived areas. Total follow‐up was 2 090 108 patient‐years (median 7 years per patient). Approximately one in four patients died during follow‐up. The most common T2DM‐related complications were coronary heart disease and heart failure. For a man diagnosed at age 50 in the most deprived quintile, with all other covariates at average values, the model predicted a remaining life expectancy of 26.7 years, 21.1 QALYs and healthcare costs of £273 k (undiscounted). For the same profile, the incremental cost‐effectiveness ratio for a hypothetical intervention reducing HbA1c by 1% was £7832 per QALY.

**Conclusions:**

We developed a T2DM policy model incorporating socio‐economic deprivation, providing estimates of life expectancy, QALYs and healthcare costs to inform health technology assessment.

## 1 Introduction

Diabetes is a leading cause of morbidity and mortality across the world. The global age‐standardised prevalence of diabetes was 6.1% with Type 2 diabetes (T2DM) making up 96% of all cases [1]. The global economic burden of diabetes is expected to increase from 1.3 trillion USD in 2015 to 2.1 trillion USD in 2030 [2]. The need for high‐quality evidence on cost‐effective interventions to improve T2DM outcomes is clear. Although randomised controlled trials often include cost‐effectiveness analyses, trial populations have limited generalisability and the majority of trials only report short‐ to medium‐term outcomes. An alternative is to develop models suitable for economic evaluation purposes using comprehensive real‐world clinical datasets.

Socio‐economic status (SES) is associated with outcomes in T2DM. Systematic reviews have shown that lower SES is associated with poorer glycaemic control [3], a higher risk of complications and increased mortality [4]. These socio‐economic gradients imply that models which omit deprivation may underestimate inequalities in long‐term outcomes and cost‐effectiveness. In a recent systematic review of T2DM decision models [5], 14 models were identified. None of these models, or any identified from our own literature search [6], includes a measure of SES (one model, published in 1997 [7], does adjust for income in the mortality component). Considering socio‐economic deprivation is important for decision‐makers who wish to mitigate health inequalities and could be achieved through the use of distributional cost‐effectiveness analysis [8]. In this study, socio‐economic position was proxied using the Scottish Index of Multiple Deprivation (SIMD) [9], which ranks small areas across seven domains (income, employment, education, health, access to services, crime and housing). SIMD was categorised into quintiles, with Quintile 1 representing the most deprived areas and Quintile 5 the least deprived. The aim of this paper is to describe and illustrate a T2DM policy model that has been developed using national linked clinical data sets and incorporates a measure of socio‐economic deprivation.

## 2 Materials and Methods

### 2.1 Model Design

This was a cohort study in which individuals newly diagnosed with T2DM between 1 January 2004 and 31 December 2020 were followed up from the date of diagnosis until death or the end of study follow‐up. Regression models using the same set of covariates were developed for mortality, complications associated with T2DM (coronary heart disease, stroke, heart failure, renal failure and amputation) and inpatient hospitalisation costs.

### 2.2 Data Sources

We used the Scottish Diabetes Research Network (SDRN) national diabetes cohort [10] which has greater than 99% coverage of the population with a diabetes diagnosis residing in Scotland to build the regression models. Estimates of disutility for the modelled complications were taken from tab. 3 of Beaudet et al.'s review [11], specifically the rows labelled ischemic heart disease, stroke, heart failure, peritoneal dialysis and amputation event. Unit costs (per bed day) were taken from Public Health Scotland's Hospital Cost Breakdown (Report code R040: Specialty costs and activity—inpatients in all specialties, excluding long stay, by specialty) and were linked to SDRN by specialty [12]. Specialties that could not be linked were removed (20% of occasions).

### 2.3 Covariates

Age, sex, SIMD, ethnicity, current smoking status, history of cardiovascular disease, body mass index (BMI), HbA1c %, systolic blood pressure (SBP), total cholesterol, high‐density lipoprotein cholesterol (HDL) and estimated glomerular filtration rate (eGFR). For the covariates listed between BMI and eGFR, we calculated the mean value across potentially multiple values measured within a time window of 6 months before and 6 months after T2DM diagnosis.

### 2.4 Modelling Remaining Life Expectancy

Gompertz survival regression with age as the timescale was used with age at T2DM as the starting time and age at death/end of follow‐up as ending time [13]. Regression modelling stratified by sex and SIMD quintiles was used to reduce the computer resources required on the server.

### 2.5 Modelling Remaining Quality Adjusted Life Expectancy

We used repeated measure logistic regression models, with yearly intervals of follow‐up as the repeated observations per patient to model the probabilities of coronary heart disease, stroke, heart failure, renal failure treated with renal replacement therapy and amputation occurring given patients' characteristics (*Note:* eGFR was not used as a covariate for renal failure models). ICD codes and other diagnosis/procedure codes used to identify these complications are shown in Supporting Information: Table A1. Each complication was modelled separately. By multiplying the survival probabilities obtained from the Gompertz regression and the probabilities of each complication at each age, we were able to get the joint probability of each complication conditional on being alive at that age.

### 2.6 Illustration of Using the Quality Adjusted Approach to Obtain Expected Dis‐Utility

Using a defined set of covariate values, we calculated the probability of none of these complications conditional on being alive, assuming that these event probabilities are additive. We then multiplied (1‐disutility) values for each complication and diabetes alone by the joint probability of developing each. We then totalled them to obtain the remaining utility at each year, and finally totalled utility estimates across years to obtain the remaining QALYs. A discount rate of 3.5% per annum [14] was applied to obtain discounted QALYs.

### 2.7 Modelling Health Care Costs

We used repeated measure linear regression to model the costs given patients' characteristics. The covariates used were the same as the covariates used to model the complications, using the quality adjusted approach above to obtain expected remaining lifetime cost. A discount rate of 3.5% per annum [14] was also applied to get discounted lifetime cost.

### 2.8 Preparing Model for Use in Economic Evaluation

As an illustration of how this model can be used as part of an economic evaluation in a wider health technology assessment, we will estimate incremental cost‐effectiveness ratios (ICERs) within sex and SIMD strata for a hypothetical treatment/intervention that reduces HbA1c by 1% (absolute reduction), reduces BMI by 5% (relative reduction) and jointly reduces HbA1c by 1% and BMI by 5%.

### 2.9 Missing Data

We used complete case analysis for all models described above (Gompertz survival models and regression models for complications and costs). We compared the baseline characteristics of individuals included in the complete case analysis with those excluded due to missing data in at least one covariate (see Supporting Information: Table A2). We also conducted multiple imputation as a sensitivity analysis using the Amelia package in R under the missing at random assumption [15]. Ten imputed datasets were generated. All models were re‐estimated separately within each imputed dataset, and pooled estimates were obtained using Rubin's rules [16].

## 3 Results

In total, we identified 269 759 people newly diagnosed with T2DM in our cohort between 1st January 2004 and 31st December 2020. Using complete case analysis reduced the size of the sample for modelling to 178 025. Table A2 in Supporting Information shows that the distributions of the covariates between complete cases and the non‐complete cases were very similar. Results from the multiple imputation analyses were also highly consistent with those from the complete case analyses across all models (Supporting Information: Tables A3, A5 and A6).

### 3.1 Baseline Demographics

The mean age at diagnosis was 63 years, almost 6 in 10 patients were male and approximately 25% of patients reside in areas belonging to the most deprived quintile (see Table 1). The highest percentage of missing data was observed for HDL.

**TABLE 1:** Demographics at time of T2DM diagnosis.

Covariates	Mean ± SD or *n* (%)
Age at diagnosis (years)	62.5 ± 11.5
Male	151 874 (56.3%)
SIMD quintile 1 (most deprived)	64 203 (23.8%)
SIMD quintile 2	62 314 (23.1%)
SIMD quintile 3	54 491 (20.2%)
SIMD quintile 4	48 557 (18.0%)
SIMD quintile 5 (least deprived)	38 036 (14.1%)
Ethnicity (White)	193 687 (71.8%)
Ethnicity (Asian)	7014 (2.6%)
Ethnicity (Other)	69 058 (25.6%)
HbA1c (mmol/mol; %)	57.6 ± 15.5; 7.4 ± 1.4
SBP (mmHg)	137.0 ± 13.7
eGFR (mL/min/1.73 m^2^)	79.6 ± 18.4
Total cholesterol (mmol/L)	4.9 ± 1.1
High‐density lipoprotein (mmol/L)	1.2 ± 0.3
Body mass index (kg/m^2^)	32.5 ± 6.7
Current smoker	59 617 (22.1%)
Previous cardiovascular disease	31 022 (11.5%)

*Note:* Continuous variables are presented as mean ± SD, and categorical variables as *n* (%); Missingness: SIMD 0.8%, HbA1c 5.6%, SBP 2.2%, eGFR 3.6%, Total cholesterol 5.0%, HDL 16.9%, BMI 8.6%, Current smoker 10.7%.

Abbreviations: eGFR: estimated glomerular filtration rate; HbA1c: glycated haemoglobin; SBP: systolic blood pressure; SD: standard deviation; SIMD: Scottish Index of Multiple Deprivation.

The total follow‐up is 2 090 108 patient‐years, with a median (IQR) follow‐up time of 7 (4–11) years per patient. During follow‐up, approximately 1 in 4 patients died (68 973/269 759)—see Table 2. For complications during follow‐up, 100 408 (37%) patients experienced at least one coronary heart disease, 31 930 (12%) heart failure, 15 172 (6%) stroke, 2452 (0.9%) amputation and 641 (0.2%) renal failure. In the same order, the mean (SD) number of complications per patient was 0.37 (1.01), 0.12 (0.49), 0.06 (0.26), 0.01 (0.11), < 0.01 (0.05), respectively.

**TABLE 2:** Total and average numbers of endpoints across follow‐up.

Endpoints	Total counts of endpoints (including repeated measurements)	Average number of endpoints per patient, mean (SD)
Death	68 973	—
Coronary heart disease	100 408	0.37 (1.01)
Stroke	15 172	0.06 (0.26)
Heart failure	31 930	0.12 (0.49)
Renal failure	641	0 (0.05)
Amputation	2452	0.01 (0.11)

### 3.2 Remaining Life Expectancy

**FIGURE 1:**
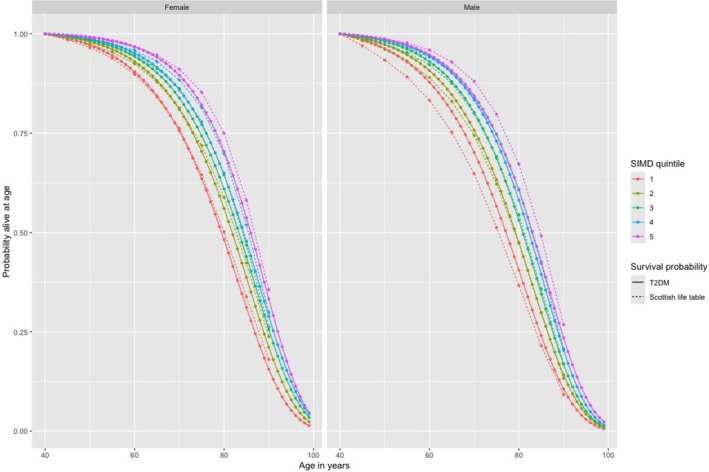
Predicted probabilities of survival from being diagnosed with T2DM at age 40 years compared to Scottish life expectancies estimated for 2014–2016.

The Gompertz regression models are shown in Supporting Information: Table A3. Key covariates when using the T2DM policy model for economic evaluation are HbA1c and SBP. As expected, across the 10 sex/SIMD stratified models, each 10 mmol/mol increase in HbA1c was associated with an estimated 8%–11% increase in the hazard of death (HR 1.08–1.11) after adjusting for all other covariates. However, for SBP the associations were against expectation; each 10 mmHg increase in SBP was associated with a lower hazard of death, with hazard ratios ranging from 0.93 to 0.96 after adjusting for all other covariates. Holding all other covariate values at their average values (see Supporting Information: Table A4), and for each sex and SIMD strata, the predicted survival probabilities for a person diagnosed with T2DM aged 40 years are shown in Figure 1 alongside survival probabilities based on Scottish life expectancies. As expected, across time, the survival probabilities are lower for people with T2DM compared to the general population, except for men in the most deprived quintile (Quintile 1). Table 3 shows the corresponding life years and QALYs across different ages of T2DM diagnosis.

**TABLE 3:** Estimated life expectancy and quality‐adjusted life years based on different diagnosis age of T2DM.

Age at diagnosis	Q1				Q2				Q3				Q4				Q5			
	M		F		M		F		M		F		M		F		M		F	
	LE	Q ALYs	LE	QALYs	LE	QALYs	LE	QALYs	LE	QALYs	LE	QALYs	LE	QALYs	LE	QALYs	LE	QALYs	LE	QALYs
40	75.6	68 (55.4)	77.8	69.8 (56)	77.6	69.5 (55.9)	80.0	71.6 (56.5)	79.2	70.9 (56.4)	81.5	72.8 (56.8)	80.6	72 (56.7)	82.5	73.6 (57.1)	81.3	72.5 (56.8)	84.1	74.9 (57.4)
50	76.7	71.1 (63.1)	78.7	72.7 (63.7)	78.5	72.5 (63.7)	80.7	74.3 (64.3)	79.8	73.6 (64.1)	82.0	75.4 (64.7)	81.1	74.6 (64.5)	83.0	76.2 (65)	81.8	75.1 (64.7)	84.5	77.3 (65.4)
60	78.7	74.9 (70.4)	80.4	76.3 (71.1)	80.1	76 (71)	82.1	77.6 (71.7)	81.1	76.8 (71.4)	83.2	78.5 (72.1)	82.2	77.6 (71.8)	84.0	79.1 (72.4)	82.9	78.2 (72)	85.2	80 (72.9)
70	82.1	79.7 (77.5)	83.3	80.7 (78.2)	83.0	80.4 (78)	84.5	81.7 (78.7)	83.6	80.9 (78.3)	85.4	82.4 (79.1)	84.3	81.5 (78.7)	86.0	82.9 (79.4)	84.9	82 (78.9)	86.7	83.4 (79.8)
80	87.0	85.8 (84.9)	87.8	86.4 (85.4)	87.5	86.2 (85.2)	88.5	87 (85.8)	87.7	86.4 (85.4)	89.0	87.4 (86.1)	88.1	86.7 (85.6)	89.4	87.7 (86.3)	88.6	87.1 (85.9)	89.7	88 (86.5)
90	93.6	93.2 (92.9)	94.0	93.5 (93.2)	93.7	93.3 (93)	94.2	93.7 (93.3)	93.7	93.3 (93)	94.5	93.9 (93.5)	93.9	93.4 (93.1)	94.6	94 (93.6)	94.2	93.6 (93.3)	94.6	94 (93.6)

*Note:* Q1–Q5: Scottish Index of Multiple Deprivation Quintile 1 to 5; M: male; F: female; LE: life expectancy; QALYs (discounted QALYs): quality adjusted life years.

### 3.3 Modelling of Secondary Events

The repeated measure logistic regression model results for each secondary complication occurring during follow‐up (CHD, stroke, heart failure, renal failure and amputation) are shown in Supporting Information: Table A5. Across the 50 sex/SIMD stratified models for each secondary event, increasing HbA1c had the expected association with the (log‐) odds of the event occurring for 96% of occasions (point estimate greater than zero in 48 models). Increasing SBP had the expected association (odds ratio > 1) on 60% of occasions, which was not the case for the 20 models with coronary heart disease and heart failure as the secondary complications.

### 3.4 Modelling of Health Care Costs

The repeated measure linear regression model results are shown in Supporting Information: Table A6. Across the 10 sex/SIMD stratified models, increasing HbA1c had the expected association of increasing health care costs coefficient estimates (ranging from £101 to £413 increase per 10 mmol/mol increase in HbA1c). After multiplying the survival probabilities obtained from the Gompertz regression and the mean predicted costs over time (holding other covariates at their average values), we obtain the estimated costs shown in Table 4. At younger ages (40 and 50 years) of T2DM diagnosis, the SIMD gradient in costs is different for males and females—for males, those residing in most versus least deprived areas accrue the lowest cost, whereas for females, those residing in most versus least deprived areas accrue the highest cost.

**TABLE 4:** Estimated costs and discounted costs (£) across based on different diagnosis age of T2DM.

Age at diagnosis	Q1		Q2		Q3		Q4		Q1	
	M	F	M	F	M	F	M	F	M	F
40	345 817 (159222)	387 978 (179095)	358 848 (161593)	345 793 (153042)	332 835 (150076)	352 857 (154434)	323 360 (145018)	339 115 (147657)	362 387 (157543)	306 802 (128089)
50	272 893 (151463)	300 254 (164632)	281 184 (152584)	289 934 (154674)	274 983 (149417)	293 424 (154205)	274 044 (147803)	290 437 (151852)	298 400 (156700)	277 792 (141818)
60	203 083 (132696)	219 392 (140730)	207 514 (132950)	227 729 (143227)	212 037 (135854)	229 146 (141925)	215 964 (137193)	232 677 (143286)	230 241 (143160)	232 424 (141109)
70	140 762 (105680)	149 213 (109948)	142 015 (105070)	164 642 (119576)	149 592 (110641)	164 871 (118159)	154 867 (113630)	170 924 (121806)	162 943 (117614)	175 875 (124382)
80	90 261 (75831)	93 444 (77359)	89 126 (74234)	107 735 (88358)	94 928 (79119)	107 095 (87000)	98 946 (81993)	112 538 (91022)	103 486 (84773)	117 022 (94440)
90	53 427 (48954)	53 164 (48345)	50 954 (46548)	62 392 (56518)	53 761 (49192)	60 891 (54916)	55 546 (50698)	64 062 (57661)	57 691 (52340)	65 887 (59366)

*Note:* Q1–Q5: Scottish Index of Multiple Deprivation Quintile 1 to 5; M: male; F: female; each cell shows total cost, with discounted cost in parentheses.

### 3.5 Economic Evaluation Illustration

In Table 5, we show estimated ICERs within sex and SIMD strata for a hypothetical treatment/intervention that reduces HbA1c by 1% (absolute reduction), reduces BMI by 5% (relative reduction) and jointly reduces HbA1c by 1% and BMI by 5%. QALY gains are shown for each age/sex/SIMD strata. In each scenario, for age 40 and age 50 at T2DM diagnosis, half or more of the ICER estimates are negative, indicating that the hypothetical interventions were dominant, resulting in QALY gains at a reduced cost. For older ages, positive ICER estimates are more common. In the 5% BMI reduction scenario, apart from six cases (SIMD quintile = 1) where QALY gains are shown as 0.01–0.02, resulting in ICERs above £20 000, all other cases are below the NICE reference threshold (£20 000–£30 000 per QALY) [17], indicating that such an intervention would be considered cost‐effective in the UK decision‐making context. For the 1% HbA1c reduction and the combined 1% HbA1c and 5% BMI reduction scenarios, all ICER estimates are well below the NICE reference threshold (the maximum estimate for 1% HbA1c reduction is £12 600 for age 90 at T2DM diagnosis, female, SIMD quintile = 3; the maximum estimate for the combined reduction is £12 315 for age 90 at diagnosis, male, SIMD quintile = 1).

**TABLE 5:** Incremental QALYs, costs (£), and ICERs for a 1% HbA1c reduction, a 5% BMI reduction and a combined 1% HbA1c and 5% BMI reduction.

Age at diagnosis	Sex	SIMD	QALY gain (disc.), 1% Hba1c	Cost difference (disc.), 1% Hba1c	ICER (disc.), 1% Hba1c	QALY gain (disc.), 5% BMI	Cost difference (disc.), 5% BMI	ICER (disc.), 5% BMI	QALY gain (disc.), 1% HbA1c + 5% BMI	Cost difference (disc.), 1% HbA1c + 5% BMI	ICER (disc.), 1% HbA1c + 5% BMI
40	M	1	0.75 (0.22)	4857 (−1229)	6498 (−5642)	0.01 (0.004)	912 (415)	77 630 (98367)	0.76 (0.22)	5786 (−812)	7611 (−3652)
40	M	2	0.64 (0.18)	3463 (−1415)	5433 (−8076)	0.06 (0.02)	852 (122)	13 199 (6578)	0.7 (0.19)	4306 (−1303)	6131 (−6735)
40	M	3	0.8 (0.21)	−4226 (−5734)	−5292 (−27 597)	0.18 (0.05)	−1547 (−1604)	−8573 (−33 418)	0.98 (0.25)	−5940 (−7401)	−6069 (−29 101)
40	M	4	0.69 (0.17)	−9028 (−7446)	−13 004 (−42 843)	0.14 (0.04)	−1195 (−1188)	−8674 (−33 858)	0.83 (0.21)	−10 355 (−8678)	−12 443 (−41 719)
40	M	5	0.58 (0.14)	−8833 (−7181)	−15 309 (−50 925)	0.17 (0.04)	466 (−577)	2669 (−13 272)	0.75 (0.18)	−8491 (−7801)	−11 300 (−42 503)
40	F	1	0.67 (0.18)	−3102 (−5134)	−4609 (−27 957)	0.1 (0.03)	689 (−155)	6719 (−5465)	0.78 (0.21)	−2470 (−5317)	−3185 (−25 148)
40	F	2	0.84 (0.21)	−4380 (−6027)	−5245 (−28 343)	0.08 (0.02)	−3102 (−1996)	−36 641 (−91 366)	0.92 (0.23)	−7634 (−8070)	−8306 (−34 532)
40	F	3	0.61 (0.15)	4677 (−288)	7714 (−1946)	0.12 (0.03)	−2511 (−1822)	−20 429 (−60 173)	0.73 (0.18)	2071 (−2142)	2845 (−12 066)
40	F	4	0.74 (0.17)	155 (−2872)	210 (−16 440)	0.07 (0.02)	−4725 (−2656)	−69 609 (−161 868)	0.8 (0.19)	−4713 (−5566)	−5858 (−29 220)
40	F	5	0.59 (0.13)	1238 (−1592)	2100 (−12 059)	0.05 (0.01)	−4013 (−2189)	−73 677 (−174 152)	0.64 (0.14)	−2864 (−3804)	−4451 (−26 373)
50	M	1	0.68 (0.26)	5355 (137)	7832 (522)	0.02 (0.01)	726 (379)	47 381 (58351)	0.7 (0.27)	6096 (520)	8715 (1938)
50	M	2	0.59 (0.22)	4021 (−257)	6789 (−1188)	0.06 (0.02)	791 (204)	12 598 (8644)	0.66 (0.24)	4806 (−63)	7333 (−262)
50	M	3	0.75 (0.26)	−1205 (−3765)	−1599 (−14 257)	0.17 (0.06)	−723 (−1113)	−4225 (−18 231)	0.93 (0.32)	−2081 (−4955)	−2249 (−15 312)
50	M	4	0.66 (0.22)	−5142 (−5493)	−7784 (−24 519)	0.13 (0.05)	−546 (−827)	−4098 (−18 038)	0.79 (0.27)	−5811 (−6376)	−7318 (−23 714)
50	M	5	0.55 (0.18)	−5411 (−5451)	−9864 (−29 939)	0.17 (0.06)	803 (−243)	4787 (−4310)	0.72 (0.24)	−4723 (−5747)	−6595 (−24 212)
50	F	1	0.63 (0.23)	−945 (−3423)	−1501 (−15 020)	0.1 (0.04)	743 (19)	7751 (552)	0.73 (0.26)	−252 (−3435)	−347 (−13 086)
50	F	2	0.79 (0.27)	−1209 (−3997)	−1525 (−14 756)	0.08 (0.03)	−2168 (−1607)	−26 702 (−57 092)	0.87 (0.3)	−3518 (−5663)	−4027 (−18 993)
50	F	3	0.58 (0.19)	5131 (612)	8854 (3197)	0.12 (0.04)	−1645 (−1423)	−13 982 (−36 302)	0.7 (0.23)	3398 (−850)	4878 (−3701)
50	F	4	0.71 (0.23)	2008 (−1521)	2840 (−6686)	0.07 (0.02)	−3511 (−2248)	−53 884 (−105 250)	0.77 (0.25)	−1638 (−3818)	−2123 (−15 389)
50	F	5	0.57 (0.18)	2625 (−605)	4583 (−3432)	0.05 (0.02)	−3000 (−1872)	−56 856 (−112 334)	0.63 (0.19)	−461 (−2507)	−737 (−13 017)
60	M	1	0.59 (0.29)	5392 (1408)	9085 (4849)	0.02 (0.01)	549 (330)	36 471 (42027)	0.61 (0.3)	5955 (1743)	9780 (5844)
60	M	2	0.52 (0.25)	4208 (870)	8034 (3513)	0.06 (0.03)	701 (276)	12 419 (10083)	0.58 (0.27)	4907 (1136)	8449 (4137)
60	M	3	0.68 (0.31)	1205 (−1586)	1775 (−5088)	0.15 (0.07)	−50 (−568)	−327 (−7900)	0.83 (0.38)	1026 (−2238)	1230 (−5851)
60	M	4	0.6 (0.27)	−1814 (−3216)	−3016 (−11 935)	0.12 (0.06)	−5 (−412)	−43 (−7447)	0.72 (0.32)	−1925 (−3692)	−2659 (−11 395)
60	M	5	0.5 (0.22)	−2451 (−3441)	−4897 (−15 642)	0.15 (0.07)	1037 (122)	6755 (1784)	0.65 (0.29)	−1512 (−3379)	−2311 (−11 767)
60	F	1	0.56 (0.26)	675 (−1662)	1209 (−6363)	0.08 (0.04)	738 (184)	8692 (4572)	0.64 (0.3)	1373 (−1509)	2132 (−5015)
60	F	2	0.72 (0.32)	1320 (−1745)	1842 (−5443)	0.07 (0.03)	−1344 (−1156)	−18 265 (−34 624)	0.79 (0.35)	−147 (−2967)	−186 (−8400)
60	F	3	0.53 (0.23)	5273 (1563)	9968 (6782)	0.11 (0.05)	−892 (−959)	−8306 (−20 340)	0.64 (0.28)	4305 (560)	6767 (2024)
60	F	4	0.65 (0.28)	3437 (21)	5286 (76)	0.06 (0.03)	−2394 (−1747)	−39 979 (−67 253)	0.71 (0.3)	920 (−1785)	1296 (−5896)
60	F	5	0.54 (0.22)	3731 (589)	6962 (2654)	0.05 (0.02)	−2049 (−1466)	−41 652 (−70 498)	0.58 (0.24)	1602 (−914)	2741 (−3773)
70	M	1	0.47 (0.29)	4852 (2266)	10 273 (7918)	0.01 (0.01)	387 (268)	30 676 (33371)	0.49 (0.29)	5253 (2540)	10 824 (8630)
70	M	2	0.42 (0.25)	3891 (1690)	9166 (6714)	0.05 (0.03)	575 (310)	12 487 (11154)	0.47 (0.28)	4468 (1994)	9480 (7135)
70	M	3	0.56 (0.33)	2635 (374)	4709 (1147)	0.13 (0.07)	381 (−69)	3006 (−926)	0.69 (0.4)	2924 (229)	4247 (572)
70	M	4	0.5 (0.29)	547 (−992)	1087 (−3442)	0.1 (0.06)	351 (−20)	3436 (−332)	0.61 (0.35)	818 (−1072)	1350 (−3088)
70	M	5	0.42 (0.24)	−283 (−1458)	−672 (−6146)	0.13 (0.07)	1101 (436)	8530 (5942)	0.55 (0.31)	744 (−1079)	1351 (−3480)
70	F	1	0.45 (0.27)	1563 (−175)	3440 (−656)	0.07 (0.04)	657 (296)	9524 (7254)	0.52 (0.31)	2192 (95)	4182 (311)
70	F	2	0.6 (0.34)	2838 (310)	4765 (917)	0.06 (0.04)	−696 (−699)	−11 381 (−19 886)	0.66 (0.37)	2045 (−454)	3112 (−1217)
70	F	3	0.44 (0.25)	4914 (2317)	11 048 (9340)	0.09 (0.05)	−323 (−496)	−3578 (−9796)	0.54 (0.3)	4532 (1778)	8467 (5964)
70	F	4	0.55 (0.3)	4128 (1445)	7478 (4764)	0.05 (0.03)	−1449 (−1198)	−28 519 (−42 335)	0.6 (0.33)	2577 (186)	4276 (560)
70	F	5	0.46 (0.25)	4268 (1759)	9188 (7003)	0.04 (0.02)	−1224 (−1002)	−28 780 (−42 861)	0.51 (0.27)	2977 (717)	5872 (2614)
80	M	1	0.33 (0.24)	3755 (2426)	11 376 (10135)	0.01 (0.01)	250 (196)	27 124 (28338)	0.34 (0.25)	4014 (2628)	11 823 (10666)
80	M	2	0.3 (0.22)	3046 (1914)	10 151 (8894)	0.03 (0.02)	416 (285)	12 696 (11977)	0.33 (0.24)	3466 (2198)	10 389 (9184)
80	M	3	0.4 (0.28)	2829 (1512)	7082 (5314)	0.09 (0.06)	509 (238)	5635 (3672)	0.49 (0.35)	3283 (1698)	6670 (4846)
80	M	4	0.36 (0.26)	1567 (543)	4316 (2118)	0.07 (0.05)	458 (229)	6209 (4371)	0.44 (0.31)	1976 (728)	4508 (2354)
80	M	5	0.31 (0.21)	779 (−23)	2544 (−109)	0.09 (0.07)	942 (577)	10 038 (8768)	0.4 (0.28)	1672 (511)	4171 (1827)
80	F	1	0.32 (0.23)	1632 (671)	5052 (2928)	0.05 (0.04)	500 (316)	10 181 (8994)	0.37 (0.26)	2115 (969)	5668 (3661)
80	F	2	0.43 (0.3)	3051 (1548)	7092 (5157)	0.04 (0.03)	−282 (−331)	−6377 (−10 632)	0.48 (0.33)	2702 (1164)	5687 (3512)
80	F	3	0.32 (0.22)	3909 (2507)	12 051 (11201)	0.07 (0.05)	−6 (−144)	−89 (−3157)	0.39 (0.27)	3863 (2329)	9888 (8646)
80	F	4	0.41 (0.28)	3776 (2214)	9296 (7965)	0.04 (0.03)	−759 (−697)	−20 278 (−26 948)	0.44 (0.3)	2944 (1463)	6634 (4818)
80	F	5	0.35 (0.24)	3889 (2403)	11 184 (10152)	0.03 (0.02)	−612 (−564)	−19 143 (−25 595)	0.38 (0.26)	3229 (1803)	8503 (6975)
90	M	1	0.18 (0.15)	2164 (1735)	11 908 (11320)	0.01 (0.005)	140 (123)	25 020 (25472)	0.19 (0.16)	2309 (1861)	12 315 (11768)
90	M	2	0.16 (0.14)	1728 (1373)	10 513 (9933)	0.02 (0.02)	234 (193)	12 648 (12302)	0.18 (0.15)	1961 (1564)	10 708 (10152)
90	M	3	0.22 (0.18)	1798 (1361)	8250 (7434)	0.05 (0.04)	340 (249)	6832 (5913)	0.27 (0.23)	2102 (1576)	7822 (6979)
90	M	4	0.2 (0.17)	1157 (804)	5843 (4843)	0.04 (0.03)	308 (230)	7592 (6752)	0.24 (0.2)	1434 (1006)	5998 (5019)
90	M	5	0.17 (0.14)	610 (343)	3678 (2482)	0.05 (0.04)	551 (437)	10 781 (10217)	0.22 (0.18)	1129 (751)	5197 (4145)
90	F	1	0.18 (0.15)	897 (601)	5100 (4084)	0.03 (0.02)	275 (218)	10 128 (9570)	0.2 (0.17)	1158 (806)	5694 (4737)
90	F	2	0.23 (0.19)	1845 (1362)	7946 (7053)	0.02 (0.02)	−124 (−144)	−5108 (−7085)	0.26 (0.21)	1679 (1182)	6544 (5537)
90	F	3	0.17 (0.14)	2192 (1764)	12 600 (12228)	0.04 (0.03)	27 (−19)	745 (−657)	0.21 (0.17)	2192 (1720)	10 454 (9899)
90	F	4	0.22 (0.18)	2198 (1712)	10 108 (9514)	0.02 (0.02)	−374 (−361)	−18 303 (−21 276)	0.24 (0.2)	1782 (1314)	7495 (6681)
90	F	5	0.19 (0.16)	2355 (1880)	12 528 (12090)	0.02 (0.01)	−274 (−269)	−15 426 (−18 120)	0.21 (0.17)	2052 (1587)	9980 (9326)

*Note:* ICERs were rounded based on the originally calculated ICERs using full‐precision cost and QALY estimates, rather than being recalculated from the rounded cost differences and QALY gains shown in the table.

Abbreviations: BMI: body mass index; disc.: discounted at 3.5% per annum; F: female; Hba1c: glycated haemoglobin; ICER: incremental cost‐effectiveness ratio; M: male; QALY: quality‐adjusted life year; SIMD: Scottish Index of Multiple Deprivation.

## 4 Discussion

This paper has introduced a T2DM policy model developed using nationwide patient level clinical data which includes a measure of socio‐economic deprivation facilitating economic evaluations that consider both efficiency and equity in informing decision making. The inputs to the model are patient demographics and biomarkers and the outputs are predicted life expectancy, quality‐adjusted life expectancy and healthcare costs.

### 4.1 Comparison With Other Studies

Here we compare our model to others reported in a recent systematic review [5]. The range of inputs used is similar, but our model includes a measure of socio‐economic deprivation [18]. Our model only includes directly incurred costs, whereas other models include both direct and indirect costs [5, 19]. Our modelling approach for life expectancy was to only use two states (alive, dead) rather than to model intermediate health states like other models [20]. The rationale for this was to simplify the process so that only one parametric survival model was used for extrapolating remaining life expectancy. Finally, our model is neither a population level Markov‐type model nor a patient level microsimulation. It is a hybrid model, with regression models fitted to individual level data, allowing model outputs for strata formed by combinations of covariate values in the linear predictor.

The QALY gains we estimate are similar to those in other literature [21]. An advantage of our model is that we can estimate QALY gains across different levels of socio‐economic deprivation.

### 4.2 Strengths and Limitations

A key strength of our approach is the use of a large, population‐based data source (> 99% coverage) that ensures our model has a low risk of selection bias. Another key strength is the inclusion of a measure of socio‐economic deprivation in our modelling framework which facilitates distributional cost‐effectiveness analysis. Other strengths are we have adhered to open science principles and have been transparent in our reporting of our coding (https://github.com/Type2DiabetesSystematicReview/he_model/tree/main).

While missing data may represent a potential source of bias, baseline characteristics were similar between included and excluded individuals and sensitivity analyses using multiple imputation yielded similar results, suggesting that this issue is unlikely to affect conclusions. Another key limitation is the presence of unexpected associations in our modelling, for example, SBP effect on risk of mortality. We rigorously assessed whether this association could be due to non‐linearity of effect (e.g., U‐ or J‐shaped associations), prescription of antihypertensive drugs or interaction effects but none of these could explain the finding. Reassuringly, our point estimates match those reported in a study of patients with T2DM after a recent acute coronary syndrome [22] with the greater sample size in our study leading to a smaller standard error and *p* value for the association. We believe the most likely explanation is residual confounding and for this reason we would replace the point estimate with one from a meta‐analysis of randomised controlled trials, which is not subject to this concern [23], before using the model to conduct economic evaluations of interventions that operate through SBP. Please note that our current economic evaluation illustration does not adjust for the unexpected associations between SBP and coronary heart disease and heart failure as secondary events—we have used the observed associations. This means that we are likely underestimating the QALY gains. Other limitations are the extent of extrapolation (only 1 in 4 people reached the final time horizon point [death]), poor measurement for some outcomes (e.g., blindness), alternative statistical modelling choices may be more valid (e.g., multivariate modelling of complications rather than individual models), important confounders may be missing (e.g., concomitant medication use) and the limited ethnicity distribution may affect generalisability of results to some settings.

### 4.3 Future Research

Our future plans are to externally validate our model using data from another population (e.g., England), while acknowledging the differences in socioeconomic deprivation measures (e.g., Index of Multiple Deprivation in England vs. SIMD in Scotland). We also plan to make the model probabilistic so we can incorporate uncertainty into our ICER estimates and include other costs beyond the health care setting.

### 4.4 Using the Model to Inform HTA

We are keen to work with other researchers and evidence users who want to use our model to inform HTA. We can modify the model if others want to make different causal assumptions [24].

In conclusion, we have developed a high‐quality T2DM policy model that can be used to inform HTA decision making around new treatments and interventions both in terms of efficiency and equity.

## Author Contributions

J.L. led the study, had management and supervision roles and drafted and edited the manuscript. L.W. conducted the data analyses and modelling, prepared tables and figures and edited the manuscript. D.M. extracted data from the SDRN server and contributed to supervision. L.A.K.B. was responsible for data acquisition, databasing and cleaning and reviewed the manuscript. All other authors reviewed and edited the manuscript.

## Funding

This research was funded by the Chief Scientist Office Scotland Health Improvement, Protection and Services Research Committee (CSO HIPS/17/26).

## Conflicts of Interest

The authors declare no conflicts of interest.

## Supporting information


**Table A1** Diagnosis and procedure codes used to identify complications.
**Table A2**. Baseline characteristics of complete cases and excluded cases (due to missing data).
**Table A3**. Gompertz regression model coefficients and CIs/*p* values.
**Table A4**. Average covariate values for each sex and SIMD strata.
**Table A5**. Repeated measure logistic models of different complications.
**Table A6**. Repeated measure linear/normal model for costs.
**Table A7**. Internal performance metrics for survival, complication, and cost models.

## Data Availability

The data used in this study are from the Scottish Diabetes Research Network (SDRN) national diabetes dataset and are not publicly available due to data governance and confidentiality restrictions. Access may be granted to qualified researchers through application to the relevant data custodians.
