# Supplementary material for: A Type 2 Diabetes Policy Model That Predicts Remaining Life Expectancy, Quality‐Adjusted Life Expectancy and Healthcare Costs for Use in Economic Evaluation, Incorporating Equity Concerns
Source: Diabetes Obes Metab. 2026 Mar 19;28(6):4772–83. doi: 10.1111/dom.70657 (PMC13146128; doi:10.1111/dom.70657)
Supplement: Supplementary file 1 — Table A1 Diagnosis and procedure codes used to identify complications. Table A2. Baseline characteristics of complete cases and excluded cases (due to missing data). Table A3. Gompertz regression model coefficients and CIs/p values. Table A4. Average covariate values for each sex and SIMD strata. Table A5. Repeated measure logistic models of different complications. Table A6. Repeated measure linear/normal model for costs. Table A7. Internal performance metrics for survival, complication, and cost models. [file DOM-28-4772-s001.docx]

Table A1. Diagnosis and procedure codes used to identify complications.

| **Complications** | **ICD-9, ICD-10 or other diagnosis/procedure codes** |
| --- | --- |
| Stroke | ICD-9: 430, 431, 434, 436 ICD-10: 160, 161, 163, 164 |
| Heart Failure | ICD-9: 402.1, 402.2, 402.4, 402.6, 402.9, 425.5, 428.1, 428.9 ICD-10: I11.0, I13.0, I13.2, I42.6, I50.0, I50.1, I50.9 |
| Coronary Heart Disease | ICD-9: 411-414 ICD-10: I20 - I25 |
| Amputation | OPCS-3: 870-875 OPCS-4: X09, X101-X104, X108, X109, X11 ICD-9: 895-897 ICD-10: S78, S88, S98, T05.3-T05.5, T13.6 |
| Renal Failure | Not applicable, captured through the Renal Registry |
|  |  |

Table A2. Baseline characteristics of complete cases and excluded cases (due to missing data).

| **Covariates** | **mean ± SD or n (%)** | |
| --- | --- | --- |
|  | Complete cases  (N = 178,025) | Excluded cases  (N = 91,734) |
| Age at diagnosis (years) | 62.5 ± 11.2 | 62.4 ± 12.0 |
| Male | 100,361 (56.4%) | 51,400 (56.0%) |
| SIMD quintile 1 (most deprived) | 43,120 (24.2%) | 20,999 (23.5%) |
| SIMD quintile 2 | 41,398 (23.3%) | 20,787 (23.2%) |
| SIMD quintile 3 | 35,929 (20.2%) | 18,617 (20.8%) |
| SIMD quintile 4 | 32,050 (18.0%) | 16,560 (18.5%) |
| SIMD quintile 5 (least deprived) | 25,528 (14.34%) | 12,503 (14.0%) |
| Ethnicity (White) | 130,653 (73.4%) | 62,990 (68.7%) |
| Ethnicity (Asian) | 4,204 (2.4%) | 2,841 (3.1%) |
| Ethnicity (Other) | 43,168 (24.3%) | 25,903 (28.2%) |
| HbA1c (mmol/mol) | 57.0 ± 14.8 | 59.1 ± 16.7 |
| SBP (mmHg) | 137.0 ±13.3 | 138.0 ±14.6 |
| eGFR (mL/min/1.73 m^2^) | 79.7 ±18.2 | 79.2 ±18.9 |
| Total cholesterol (mmol/L) | 4.9 ± 1.1 | 4.9 ± 1.1 |
| High-Density Lipoprotein (mmol/L) | 1.2 ± 0.3 | 1.2 ± 0.3 |
| Body Mass Index (kg/m2) | 32.6 ± 6.6 | 32.4 ± 6.9 |
| Current smoker | 39,031 (21.9%) | 14,340 (22.8%) |
| Previous cardiovascular disease | 21,101 (11.9%) | 9,956 (10.9%) |
| Continuous variables are presented as mean ± SD, and categorical variables as n (%), where percentages are calculated among individuals with non-missing values for the respective variable; SD: standard deviation; SIMD: Scottish Index of Multiple Deprivation; HbA1c: Glycated haemoglobin; SBP: systolic blood pressure; eGFR: estimated Glomerular Filtration Rate. | | |

Table A3. Gompertz regression model coefficients and CIs / p-values

| Sex | SIMD | Parameter | HR (95%CI) | P value | HR imputed (95%CI) | P value (imputed) |
| --- | --- | --- | --- | --- | --- | --- |
| M | 1 | shape | 0.09 (0.09, 0.09) | NA | 0.09 (0.09, 0.09) | NA |
| M | 1 | rate | 0 (0, 0) | NA | 0 (-0.39, 0.39) | NA |
| M | 1 | total_chol | 0.98 (0.96, 1.01) | 0.125 | 0.99 (0.97, 1.02) | 0.611 |
| M | 1 | current_smoker | 1.78 (1.68, 1.88) | <0.001 | 1.72 (1.64, 1.8) | <0.001 |
| M | 1 | hdl | 1.25 (1.15, 1.36) | <0.001 | 1.22 (1.17, 1.28) | <0.001 |
| M | 1 | I(sbp/10) | 0.95 (0.93, 0.97) | <0.001 | 0.95 (0.94, 0.97) | <0.001 |
| M | 1 | ethnicasian | 0.45 (0.33, 0.62) | <0.001 | 0.82 (0.68, 0.98) | 0.028 |
| M | 1 | ethnicother | 1.34 (1.26, 1.42) | <0.001 | 1.05 (0.93, 1.17) | 0.434 |
| M | 1 | I(egfr/10) | 1 (0.98, 1.02) | 0.368 | 0.99 (0.98, 1.01) | 0.495 |
| M | 1 | I(hba1c/10) | 1.09 (1.07, 1.11) | <0.001 | 1.09 (1.07, 1.1) | <0.001 |
| M | 1 | bmi | 1 (1, 1.01) | 0.309 | 1 (1, 1) | 0.672 |
| M | 1 | cv_disease | 1.33 (1.25, 1.42) | <0.001 | 1.37 (1.3, 1.45) | <0.001 |
| M | 1 | simd_b | 0.88 (0.84, 0.93) | <0.001 | 0.91 (0.87, 0.95) | <0.001 |
| M | 2 | shape | 0.09 (0.09, 0.1) | NA | 0.09 (0.09, 0.1) | NA |
| M | 2 | rate | 0 (0, 0) | NA | 0 (-0.4, 0.4) | NA |
| M | 2 | total_chol | 0.98 (0.95, 1.01) | 0.145 | 1 (0.97, 1.02) | 0.936 |
| M | 2 | current_smoker | 1.93 (1.81, 2.05) | <0.001 | 1.81 (1.71, 1.9) | <0.001 |
| M | 2 | hdl | 1.29 (1.18, 1.42) | <0.001 | 1.16 (1.11, 1.21) | <0.001 |
| M | 2 | I(sbp/10) | 0.94 (0.92, 0.96) | <0.001 | 0.95 (0.93, 0.97) | <0.001 |
| M | 2 | ethnicasian | 0.33 (0.22, 0.49) | <0.001 | 0.82 (0.69, 0.96) | 0.017 |
| M | 2 | ethnicother | 1.37 (1.3, 1.46) | <0.001 | 1.04 (0.89, 1.22) | 0.581 |
| M | 2 | I(egfr/10) | 0.98 (0.96, 1) | 0.026 | 0.97 (0.96, 0.99) | <0.001 |
| M | 2 | I(hba1c/10) | 1.08 (1.06, 1.1) | <0.001 | 1.08 (1.06, 1.09) | <0.001 |
| M | 2 | bmi | 1.01 (1, 1.01) | 0.025 | 1 (1, 1.01) | 0.121 |
| M | 2 | cv_disease | 1.37 (1.29, 1.47) | <0.001 | 1.39 (1.31, 1.46) | <0.001 |
| M | 2 | simd_b | 0.99 (0.94, 1.04) | 0.315 | 0.96 (0.92, 1) | 0.051 |
| M | 3 | shape | 0.1 (0.1, 0.11) | NA | 0.1 (0.1, 0.1) | NA |
| M | 3 | rate | 0 (0, 0) | NA | 0 (-0.44, 0.44) | NA |
| M | 3 | total_chol | 1.02 (0.99, 1.05) | 0.136 | 1.01 (0.98, 1.04) | 0.448 |
| M | 3 | current_smoker | 1.82 (1.7, 1.95) | <0.001 | 1.73 (1.64, 1.84) | <0.001 |
| M | 3 | hdl | 1.11 (1.01, 1.23) | 0.017 | 1.14 (1.09, 1.2) | <0.001 |
| M | 3 | I(sbp/10) | 0.95 (0.93, 0.97) | <0.001 | 0.94 (0.93, 0.96) | <0.001 |
| M | 3 | ethnicasian | 0.64 (0.46, 0.89) | 0.004 | 0.93 (0.77, 1.12) | 0.434 |
| M | 3 | ethnicother | 1.31 (1.23, 1.39) | <0.001 | 1.15 (1.01, 1.32) | 0.041 |
| M | 3 | I(egfr/10) | 0.97 (0.95, 0.99) | 0.002 | 0.98 (0.96, 1) | 0.013 |
| M | 3 | I(hba1c/10) | 1.1 (1.08, 1.13) | <0.001 | 1.1 (1.08, 1.11) | <0.001 |
| M | 3 | bmi | 1.02 (1.01, 1.02) | <0.001 | 1.01 (1.01, 1.02) | <0.001 |
| M | 3 | cv_disease | 1.37 (1.27, 1.47) | <0.001 | 1.4 (1.32, 1.48) | <0.001 |
| M | 3 | simd_b | 0.92 (0.87, 0.98) | 0.002 | 0.94 (0.9, 0.98) | 0.006 |
| M | 4 | shape | 0.11 (0.1, 0.11) | NA | 0.1 (0.1, 0.11) | NA |
| M | 4 | rate | 0 (0, 0) | NA | 0 (-0.48, 0.48) | NA |
| M | 4 | total_chol | 0.99 (0.96, 1.03) | 0.335 | 0.99 (0.96, 1.02) | 0.539 |
| M | 4 | current_smoker | 1.91 (1.76, 2.06) | <0.001 | 1.76 (1.65, 1.89) | <0.001 |
| M | 4 | hdl | 1.14 (1.03, 1.27) | 0.006 | 1.17 (1.11, 1.24) | <0.001 |
| M | 4 | I(sbp/10) | 0.94 (0.92, 0.97) | <0.001 | 0.95 (0.93, 0.97) | <0.001 |
| M | 4 | ethnicasian | 0.75 (0.53, 1.08) | 0.06 | 0.96 (0.81, 1.14) | 0.653 |
| M | 4 | ethnicother | 1.3 (1.22, 1.39) | <0.001 | 1.14 (1.02, 1.27) | 0.019 |
| M | 4 | I(egfr/10) | 0.97 (0.95, 0.99) | 0.003 | 0.97 (0.95, 0.99) | 0.002 |
| M | 4 | I(hba1c/10) | 1.09 (1.07, 1.12) | <0.001 | 1.1 (1.09, 1.12) | <0.001 |
| M | 4 | bmi | 1.01 (1.01, 1.02) | <0.001 | 1.01 (1, 1.01) | 0.01 |
| M | 4 | cv_disease | 1.42 (1.32, 1.54) | <0.001 | 1.43 (1.34, 1.53) | <0.001 |
| M | 4 | simd_b | 0.95 (0.9, 1.01) | 0.061 | 0.96 (0.92, 1.01) | 0.133 |
| M | 5 | shape | 0.11 (0.1, 0.11) | NA | 0.1 (0.1, 0.11) | NA |
| M | 5 | rate | 0 (0, 0) | NA | 0 (-0.55, 0.55) | NA |
| M | 5 | total_chol | 0.99 (0.95, 1.03) | 0.339 | 1.01 (0.98, 1.04) | 0.603 |
| M | 5 | current_smoker | 1.8 (1.63, 1.98) | <0.001 | 1.68 (1.54, 1.83) | <0.001 |
| M | 5 | hdl | 1.32 (1.18, 1.48) | <0.001 | 1.11 (1.05, 1.18) | <0.001 |
| M | 5 | I(sbp/10) | 0.96 (0.93, 0.99) | 0.002 | 0.96 (0.93, 0.98) | <0.001 |
| M | 5 | ethnicasian | 0.54 (0.4, 0.72) | <0.001 | 0.81 (0.69, 0.94) | 0.007 |
| M | 5 | ethnicother | 1.29 (1.2, 1.39) | <0.001 | 1.09 (0.95, 1.25) | 0.228 |
| M | 5 | I(egfr/10) | 0.98 (0.95, 1) | 0.052 | 0.97 (0.95, 0.99) | 0.006 |
| M | 5 | I(hba1c/10) | 1.08 (1.05, 1.1) | <0.001 | 1.1 (1.08, 1.12) | <0.001 |
| M | 5 | bmi | 1.02 (1.01, 1.02) | <0.001 | 1.01 (1, 1.02) | 0.007 |
| M | 5 | cv_disease | 1.4 (1.28, 1.54) | <0.001 | 1.42 (1.32, 1.53) | <0.001 |
| M | 5 | simd_b | 1 (0.93, 1.07) | 0.458 | 0.98 (0.92, 1.03) | 0.41 |
| F | 1 | shape | 0.09 (0.09, 0.1) | NA | 0.09 (0.09, 0.09) | NA |
| F | 1 | rate | 0 (0, 0) | NA | 0 (-0.39, 0.39) | NA |
| F | 1 | total_chol | 0.98 (0.95, 1.01) | 0.07 | 0.99 (0.96, 1.01) | 0.197 |
| F | 1 | current_smoker | 1.89 (1.78, 2.01) | <0.001 | 1.83 (1.74, 1.93) | <0.001 |
| F | 1 | hdl | 1.09 (1, 1.18) | 0.024 | 1.14 (1.09, 1.19) | <0.001 |
| F | 1 | I(sbp/10) | 0.95 (0.93, 0.97) | <0.001 | 0.96 (0.94, 0.97) | <0.001 |
| F | 1 | ethnicasian | 0.58 (0.38, 0.89) | 0.007 | 0.93 (0.78, 1.12) | 0.461 |
| F | 1 | ethnicother | 1.36 (1.27, 1.45) | <0.001 | 1.12 (0.96, 1.31) | 0.136 |
| F | 1 | I(egfr/10) | 0.98 (0.96, 1) | 0.01 | 0.97 (0.96, 0.99) | <0.001 |
| F | 1 | I(hba1c/10) | 1.08 (1.06, 1.1) | <0.001 | 1.08 (1.06, 1.09) | <0.001 |
| F | 1 | bmi | 1.01 (1, 1.01) | <0.001 | 1.01 (1, 1.01) | 0.004 |
| F | 1 | cv_disease | 1.4 (1.3, 1.5) | <0.001 | 1.45 (1.37, 1.53) | <0.001 |
| F | 1 | simd_b | 0.96 (0.91, 1.01) | 0.075 | 0.95 (0.91, 1) | 0.033 |
| F | 2 | shape | 0.1 (0.09, 0.1) | NA | 0.09 (0.09, 0.1) | NA |
| F | 2 | rate | 0 (0, 0) | NA | 0 (-0.43, 0.43) | NA |
| F | 2 | total_chol | 0.98 (0.95, 1.01) | 0.054 | 0.99 (0.97, 1.01) | 0.363 |
| F | 2 | current_smoker | 2 (1.87, 2.14) | <0.001 | 1.85 (1.75, 1.96) | <0.001 |
| F | 2 | hdl | 1.08 (0.99, 1.17) | 0.04 | 1.1 (1.05, 1.16) | <0.001 |
| F | 2 | I(sbp/10) | 0.95 (0.93, 0.97) | <0.001 | 0.95 (0.94, 0.97) | <0.001 |
| F | 2 | ethnicasian | 0.61 (0.41, 0.92) | 0.01 | 1.02 (0.84, 1.24) | 0.838 |
| F | 2 | ethnicother | 1.27 (1.19, 1.35) | <0.001 | 1.05 (0.92, 1.2) | 0.47 |
| F | 2 | I(egfr/10) | 0.96 (0.95, 0.98) | <0.001 | 0.96 (0.95, 0.98) | <0.001 |
| F | 2 | I(hba1c/10) | 1.11 (1.09, 1.13) | <0.001 | 1.11 (1.09, 1.13) | <0.001 |
| F | 2 | bmi | 1.01 (1, 1.01) | 0.003 | 1 (1, 1.01) | 0.03 |
| F | 2 | cv_disease | 1.48 (1.37, 1.6) | <0.001 | 1.52 (1.43, 1.61) | <0.001 |
| F | 2 | simd_b | 0.95 (0.9, 1.01) | 0.039 | 0.95 (0.91, 0.99) | 0.024 |
| F | 3 | shape | 0.1 (0.1, 0.1) | NA | 0.1 (0.1, 0.1) | NA |
| F | 3 | rate | 0 (0, 0) | NA | 0 (-0.47, 0.47) | NA |
| F | 3 | total_chol | 0.99 (0.95, 1.02) | 0.244 | 1 (0.98, 1.03) | 0.891 |
| F | 3 | current_smoker | 2.26 (2.09, 2.45) | <0.001 | 2.01 (1.88, 2.16) | <0.001 |
| F | 3 | hdl | 1.04 (0.95, 1.15) | 0.184 | 1.11 (1.06, 1.17) | <0.001 |
| F | 3 | I(sbp/10) | 0.95 (0.92, 0.97) | <0.001 | 0.95 (0.94, 0.97) | <0.001 |
| F | 3 | ethnicasian | 0.73 (0.46, 1.19) | 0.103 | 1.05 (0.85, 1.29) | 0.676 |
| F | 3 | ethnicother | 1.33 (1.24, 1.43) | <0.001 | 1.04 (0.93, 1.16) | 0.474 |
| F | 3 | I(egfr/10) | 0.95 (0.93, 0.97) | <0.001 | 0.94 (0.93, 0.96) | <0.001 |
| F | 3 | I(hba1c/10) | 1.08 (1.05, 1.1) | <0.001 | 1.09 (1.07, 1.11) | <0.001 |
| F | 3 | bmi | 1.01 (1, 1.02) | <0.001 | 1.01 (1, 1.01) | 0.007 |
| F | 3 | cv_disease | 1.46 (1.33, 1.59) | <0.001 | 1.53 (1.43, 1.64) | <0.001 |
| F | 3 | simd_b | 0.96 (0.91, 1.03) | 0.134 | 0.95 (0.9, 1) | 0.04 |
| F | 4 | shape | 0.1 (0.1, 0.11) | NA | 0.1 (0.1, 0.11) | NA |
| F | 4 | rate | 0 (0, 0) | NA | 0 (-0.51, 0.51) | NA |
| F | 4 | total_chol | 0.97 (0.94, 1.01) | 0.083 | 0.99 (0.96, 1.02) | 0.5 |
| F | 4 | current_smoker | 2.03 (1.85, 2.23) | <0.001 | 1.89 (1.74, 2.06) | <0.001 |
| F | 4 | hdl | 1.05 (0.94, 1.16) | 0.198 | 1.13 (1.07, 1.2) | <0.001 |
| F | 4 | I(sbp/10) | 0.93 (0.91, 0.96) | <0.001 | 0.93 (0.91, 0.95) | <0.001 |
| F | 4 | ethnicasian | 0.64 (0.41, 1) | 0.026 | 0.99 (0.79, 1.24) | 0.933 |
| F | 4 | ethnicother | 1.35 (1.26, 1.46) | <0.001 | 1.05 (0.91, 1.21) | 0.485 |
| F | 4 | I(egfr/10) | 0.95 (0.92, 0.97) | <0.001 | 0.94 (0.93, 0.96) | <0.001 |
| F | 4 | I(hba1c/10) | 1.1 (1.07, 1.13) | <0.001 | 1.1 (1.08, 1.12) | <0.001 |
| F | 4 | bmi | 1.01 (1, 1.01) | 0.038 | 1 (1, 1.01) | 0.1 |
| F | 4 | cv_disease | 1.44 (1.31, 1.59) | <0.001 | 1.46 (1.35, 1.57) | <0.001 |
| F | 4 | simd_b | 0.92 (0.86, 0.98) | 0.008 | 0.93 (0.88, 0.99) | 0.014 |
| F | 5 | shape | 0.11 (0.11, 0.12) | NA | 0.11 (0.11, 0.11) | NA |
| F | 5 | rate | 0 (0, 0) | NA | 0 (-0.62, 0.62) | NA |
| F | 5 | total_chol | 1.01 (0.97, 1.06) | 0.301 | 1.03 (1, 1.07) | 0.064 |
| F | 5 | current_smoker | 2.02 (1.8, 2.26) | <0.001 | 1.83 (1.64, 2.04) | <0.001 |
| F | 5 | hdl | 0.96 (0.86, 1.08) | 0.24 | 1.1 (1.03, 1.18) | 0.006 |
| F | 5 | I(sbp/10) | 0.95 (0.92, 0.98) | <0.001 | 0.93 (0.91, 0.96) | <0.001 |
| F | 5 | ethnicasian | 0.45 (0.28, 0.71) | <0.001 | 0.86 (0.7, 1.05) | 0.141 |
| F | 5 | ethnicother | 1.24 (1.13, 1.35) | <0.001 | 1 (0.87, 1.16) | 0.957 |
| F | 5 | I(egfr/10) | 0.96 (0.93, 0.98) | <0.001 | 0.96 (0.93, 0.98) | <0.001 |
| F | 5 | I(hba1c/10) | 1.09 (1.06, 1.12) | <0.001 | 1.09 (1.07, 1.12) | <0.001 |
| F | 5 | bmi | 1 (1, 1.01) | 0.094 | 1 (1, 1.01) | 0.18 |
| F | 5 | cv_disease | 1.45 (1.29, 1.62) | <0.001 | 1.53 (1.4, 1.67) | <0.001 |
| F | 5 | simd_b | 0.99 (0.91, 1.07) | 0.356 | 1 (0.94, 1.06) | 0.963 |
| HR: hazard ratio; HRs are from the complete-case analysis; HR imputed (95% CI) and P value (imputed) represent pooled estimates based on Rubin’s rules across 10 imputed datasets; HRs are reported for covariates only. Gompertz shape and rate parameters are distributional parameters and are therefore presented on their natural scale; CI: confidence interval; SIMD: Scottish Index of Multiple Deprivation; M: Male; F: Female; total_chol: Total cholesterol; hdl: High-density lipoprotein cholesterol; I(sbp/10): Systolic blood pressure (per 10 mmHg increase); ethnicasian: Ethnicity – Asian; ethnicother: Ethnicity – Other; I(egfr/10): Estimated glomerular filtration rate (per 10 mL/min/1.73m^2^ increase); I(hba1c/10): Glycated haemoglobin (HbA1c) (per 10 mmol/mol increase); bmi: Body mass index; cv_disease: History of cardiovascular disease; simd_b: Binary indicator for each SIMD quintile - coded as 0 for the lower decile (e.g., decile 1) and 1 for the higher decile (e.g., decile 2), as the original SIMD variable is in deciles. | | | | | | |

Table A4. Average covariate values for each sex and SIMD strata.

| Stratified by     Covariates | M, Q1 | M, Q 2 | M, Q3 | M, Q 4 | M, Q 5 | F, Q 1 | F, Q 2 | F, Q 3 | F, Q 4 | F, Q 5 |
| --- | --- | --- | --- | --- | --- | --- | --- | --- | --- | --- |
| SBP (mmHg) | 136.3 | 136.9 | 137.3 | 137.6 | 137.2 | 136.6 | 137.6 | 138.2 | 138.4 | 138.6 |
| eGFR (mL/min/1.73 m^2^) | 84.3 | 82.4 | 81.5 | 81.3 | 80.9 | 77.7 | 76 | 75.7 | 75.7 | 75.5 |
| HbA1c (mmol/mol) | 59.8 | 58.9 | 58 | 57.5 | 56.7 | 57.7 | 57 | 56.3 | 55.9 | 55.6 |
| Total cholesterol (mmol/L) | 4.8 | 4.7 | 4.7 | 4.7 | 4.7 | 5.1 | 5.1 | 5.1 | 5.1 | 5.1 |
| High-Density Lipoprotein (mmol/L) | 1.1 | 1.1 | 1.1 | 1.1 | 1.1 | 1.2 | 1.3 | 1.3 | 1.3 | 1.3 |
| Body Mass Index (kg/m^2^) | 32.4 | 32.2 | 31.9 | 31.6 | 30.9 | 34.3 | 33.7 | 33.3 | 32.7 | 31.8 |
| Current smoker | 31% | 25% | 21% | 17% | 13% | 32% | 24% | 20% | 15% | 12% |
| Previous cardiovascular disease | 14% | 14% | 13% | 12% | 11% | 11% | 10% | 9% | 8% | 8% |
| Higher SIMD decile within quintile (%) | 50% | 49% | 49% | 48% | 45% | 50% | 48% | 48% | 48% | 44% |
| Ethnicity (Asian) | 3% | 2% | 3% | 2% | 4% | 2% | 2% | 2% | 3% | 4% |
| Ethnicity (Other) | 21% | 25% | 28% | 28% | 29% | 21% | 25% | 28% | 28% | 29% |
| Ethnicity (White) | 77% | 72% | 70% | 70% | 67% | 78% | 73% | 70% | 70% | 67% |
| Average values for numerical and categorical covariates are displayed as mean and %; SIMD: Scottish Index of Multiple Deprivation; Higher SIMD decile within quintile (%) indicates the higher decile of each SIMD quintile (e.g., decile 2 within quintile 1), given that SIMD is originally defined in deciles. Q1-Q5: SIMD Quintile 1- 5; M: Male; F: Female. | | | | | | | | | | |

Table A5. Repeated measure logistic models of different complications.

| Sex | SIMD | Parameter | OR (95%CI) | P value | OR imputed (95%CI) | P value (imputed) | Event |
| --- | --- | --- | --- | --- | --- | --- | --- |
| M | 1 | (Intercept) | -12.89 (-15.11, -10.68) | NA | -11.63 (-13.33, -9.93) | NA | Amputation |
| M | 1 | agenow10 | 3.53 (2.7, 4.62) | <0.001 | 3.13 (2.56, 3.83) | <0.001 | Amputation |
| M | 1 | agedx10 | 0.37 (0.28, 0.48) | <0.001 | 0.38 (0.31, 0.47) | <0.001 | Amputation |
| M | 1 | simd_b | 0.86 (0.68, 1.09) | 0.212 | 1.02 (0.85, 1.22) | 0.842 | Amputation |
| M | 1 | current_smoker | 1.59 (1.24, 2.03) | <0.001 | 1.52 (1.24, 1.87) | <0.001 | Amputation |
| M | 1 | total_chol | 1.08 (0.97, 1.21) | 0.17 | 1.03 (0.95, 1.12) | 0.468 | Amputation |
| M | 1 | hdl | 0.72 (0.46, 1.12) | 0.146 | 1.06 (0.87, 1.28) | 0.586 | Amputation |
| M | 1 | I(sbp/10) | 1.25 (1.15, 1.36) | <0.001 | 1.19 (1.11, 1.27) | <0.001 | Amputation |
| M | 1 | ethnicasian | 0.14 (0.02, 0.98) | 0.047 | 0.45 (0.21, 1.01) | 0.052 | Amputation |
| M | 1 | ethnicother | 0.68 (0.47, 0.99) | 0.043 | 0.67 (0.36, 1.25) | 0.208 | Amputation |
| M | 1 | I(egfr/10) | 1.1 (1, 1.21) | 0.047 | 1.09 (1.01, 1.17) | 0.019 | Amputation |
| M | 1 | I(hba1c/10) | 1.24 (1.16, 1.32) | <0.001 | 1.26 (1.21, 1.32) | <0.001 | Amputation |
| M | 1 | cv_disease | 1.64 (1.2, 2.26) | 0.002 | 1.6 (1.25, 2.05) | <0.001 | Amputation |
| M | 1 | bmi | 0.96 (0.94, 0.98) | <0.001 | 0.95 (0.94, 0.97) | <0.001 | Amputation |
| M | 2 | (Intercept) | -11.88 (-14.45, -9.32) | NA | -11.15 (-13.1, -9.21) | NA | Amputation |
| M | 2 | agenow10 | 3.51 (2.59, 4.76) | <0.001 | 2.23 (1.77, 2.79) | <0.001 | Amputation |
| M | 2 | agedx10 | 0.38 (0.28, 0.53) | <0.001 | 0.52 (0.41, 0.66) | <0.001 | Amputation |
| M | 2 | simd_b | 1.35 (1.04, 1.76) | 0.025 | 1.23 (1.01, 1.5) | 0.043 | Amputation |
| M | 2 | current_smoker | 1.99 (1.5, 2.63) | <0.001 | 1.79 (1.42, 2.25) | <0.001 | Amputation |
| M | 2 | total_chol | 0.99 (0.86, 1.14) | 0.876 | 0.98 (0.88, 1.09) | 0.699 | Amputation |
| M | 2 | hdl | 1.45 (0.92, 2.27) | 0.106 | 1.33 (1.08, 1.63) | 0.006 | Amputation |
| M | 2 | I(sbp/10) | 1 (0.91, 1.11) | 0.953 | 1.1 (1.02, 1.19) | 0.011 | Amputation |
| M | 2 | ethnicasian | 0.48 (0.12, 1.95) | 0.305 | 0.52 (0.17, 1.56) | 0.233 | Amputation |
| M | 2 | ethnicother | 1.21 (0.88, 1.65) | 0.235 | 0.68 (0.35, 1.31) | 0.245 | Amputation |
| M | 2 | I(egfr/10) | 1.15 (1.03, 1.28) | 0.01 | 1.09 (1, 1.19) | 0.062 | Amputation |
| M | 2 | I(hba1c/10) | 1.19 (1.1, 1.28) | <0.001 | 1.24 (1.17, 1.31) | <0.001 | Amputation |
| M | 2 | cv_disease | 1.86 (1.32, 2.62) | <0.001 | 1.91 (1.47, 2.48) | <0.001 | Amputation |
| M | 2 | bmi | 0.98 (0.96, 1.01) | 0.199 | 0.98 (0.96, 0.99) | 0.013 | Amputation |
| M | 3 | (Intercept) | -12.46 (-15.03, -9.89) | NA | -13.08 (-15.13, -11.03) | NA | Amputation |
| M | 3 | agenow10 | 2.81 (2.04, 3.88) | <0.001 | 2.3 (1.79, 2.95) | <0.001 | Amputation |
| M | 3 | agedx10 | 0.49 (0.35, 0.69) | <0.001 | 0.61 (0.47, 0.79) | <0.001 | Amputation |
| M | 3 | simd_b | 0.91 (0.7, 1.2) | 0.523 | 0.87 (0.7, 1.07) | 0.19 | Amputation |
| M | 3 | current_smoker | 1.57 (1.15, 2.15) | 0.005 | 1.82 (1.42, 2.33) | <0.001 | Amputation |
| M | 3 | total_chol | 1 (0.86, 1.16) | 0.98 | 0.95 (0.85, 1.06) | 0.366 | Amputation |
| M | 3 | hdl | 0.77 (0.45, 1.32) | 0.341 | 1.09 (0.86, 1.37) | 0.493 | Amputation |
| M | 3 | I(sbp/10) | 1.08 (0.97, 1.2) | 0.169 | 1.12 (1.03, 1.21) | 0.007 | Amputation |
| M | 3 | ethnicasian | 0 | 0.961 | 0.31 (0.07, 1.36) | 0.117 | Amputation |
| M | 3 | ethnicother | 0.99 (0.72, 1.38) | 0.972 | 0.78 (0.42, 1.47) | 0.442 | Amputation |
| M | 3 | I(egfr/10) | 1 (0.9, 1.11) | 0.964 | 1.08 (0.99, 1.18) | 0.08 | Amputation |
| M | 3 | I(hba1c/10) | 1.41 (1.32, 1.5) | <0.001 | 1.4 (1.33, 1.47) | <0.001 | Amputation |
| M | 3 | cv_disease | 1.78 (1.24, 2.56) | 0.002 | 2.15 (1.63, 2.85) | <0.001 | Amputation |
| M | 3 | bmi | 1 (0.98, 1.03) | 0.79 | 0.99 (0.96, 1.01) | 0.23 | Amputation |
| M | 4 | (Intercept) | -11.52 (-14.21, -8.84) | NA | -10.9 (-13.13, -8.66) | NA | Amputation |
| M | 4 | agenow10 | 2.91 (2.11, 4.02) | <0.001 | 2.15 (1.67, 2.77) | <0.001 | Amputation |
| M | 4 | agedx10 | 0.54 (0.39, 0.75) | <0.001 | 0.62 (0.48, 0.8) | <0.001 | Amputation |
| M | 4 | simd_b | 0.77 (0.59, 1.02) | 0.067 | 0.97 (0.78, 1.2) | 0.753 | Amputation |
| M | 4 | current_smoker | 1.85 (1.34, 2.55) | <0.001 | 1.73 (1.31, 2.28) | <0.001 | Amputation |
| M | 4 | total_chol | 0.92 (0.79, 1.07) | 0.29 | 0.86 (0.76, 0.97) | 0.012 | Amputation |
| M | 4 | hdl | 1.34 (0.86, 2.1) | 0.197 | 1.17 (0.92, 1.48) | 0.199 | Amputation |
| M | 4 | I(sbp/10) | 1.07 (0.96, 1.18) | 0.234 | 1.16 (1.07, 1.26) | <0.001 | Amputation |
| M | 4 | ethnicasian | 0 | 0.961 | 0.36 (0.07, 1.76) | 0.194 | Amputation |
| M | 4 | ethnicother | 0.95 (0.69, 1.32) | 0.775 | 1 (0.57, 1.76) | 0.993 | Amputation |
| M | 4 | I(egfr/10) | 1.1 (0.99, 1.23) | 0.077 | 1.06 (0.97, 1.16) | 0.223 | Amputation |
| M | 4 | I(hba1c/10) | 1.28 (1.19, 1.38) | <0.001 | 1.31 (1.23, 1.39) | <0.001 | Amputation |
| M | 4 | cv_disease | 1.54 (1.05, 2.26) | 0.027 | 1.66 (1.22, 2.25) | 0.001 | Amputation |
| M | 4 | bmi | 0.94 (0.92, 0.97) | <0.001 | 0.95 (0.92, 0.97) | <0.001 | Amputation |
| M | 5 | (Intercept) | -15.65 (-19.09, -12.21) | NA | -14.66 (-17.49, -11.83) | NA | Amputation |
| M | 5 | agenow10 | 3.81 (2.56, 5.67) | <0.001 | 2.98 (2.15, 4.12) | <0.001 | Amputation |
| M | 5 | agedx10 | 0.46 (0.31, 0.69) | <0.001 | 0.55 (0.39, 0.76) | <0.001 | Amputation |
| M | 5 | simd_b | 0.62 (0.43, 0.9) | 0.011 | 0.76 (0.57, 1.01) | 0.063 | Amputation |
| M | 5 | current_smoker | 2.53 (1.7, 3.77) | <0.001 | 2.18 (1.46, 3.25) | <0.001 | Amputation |
| M | 5 | total_chol | 1.09 (0.91, 1.3) | 0.353 | 0.99 (0.85, 1.15) | 0.875 | Amputation |
| M | 5 | hdl | 1.35 (0.76, 2.42) | 0.309 | 0.72 (0.51, 1.02) | 0.063 | Amputation |
| M | 5 | I(sbp/10) | 1.14 (1.01, 1.3) | 0.037 | 1.18 (1.06, 1.31) | 0.002 | Amputation |
| M | 5 | ethnicasian | 0 | 0.97 | 0.34 (0.07, 1.66) | 0.175 | Amputation |
| M | 5 | ethnicother | 0.91 (0.61, 1.36) | 0.641 | 0.98 (0.46, 2.08) | 0.966 | Amputation |
| M | 5 | I(egfr/10) | 1.1 (0.96, 1.26) | 0.155 | 1.16 (1.04, 1.31) | 0.011 | Amputation |
| M | 5 | I(hba1c/10) | 1.32 (1.2, 1.46) | <0.001 | 1.29 (1.2, 1.4) | <0.001 | Amputation |
| M | 5 | cv_disease | 1.3 (0.74, 2.26) | 0.359 | 1.45 (0.94, 2.25) | 0.094 | Amputation |
| M | 5 | bmi | 0.98 (0.94, 1.01) | 0.23 | 0.98 (0.95, 1.01) | 0.173 | Amputation |
| F | 1 | (Intercept) | -10.54 (-13.86, -7.23) | NA | -9.95 (-12.46, -7.45) | NA | Amputation |
| F | 1 | agenow10 | 1.52 (0.98, 2.36) | 0.063 | 1.84 (1.33, 2.53) | <0.001 | Amputation |
| F | 1 | agedx10 | 0.71 (0.45, 1.13) | 0.149 | 0.49 (0.35, 0.69) | <0.001 | Amputation |
| F | 1 | simd_b | 0.93 (0.64, 1.34) | 0.69 | 1.2 (0.91, 1.59) | 0.201 | Amputation |
| F | 1 | current_smoker | 2.26 (1.53, 3.35) | <0.001 | 2.06 (1.53, 2.77) | <0.001 | Amputation |
| F | 1 | total_chol | 1.1 (0.93, 1.32) | 0.269 | 1.1 (0.97, 1.25) | 0.141 | Amputation |
| F | 1 | hdl | 1.06 (0.6, 1.86) | 0.842 | 1.04 (0.77, 1.4) | 0.813 | Amputation |
| F | 1 | I(sbp/10) | 1.19 (1.05, 1.36) | 0.008 | 1.17 (1.06, 1.3) | 0.001 | Amputation |
| F | 1 | ethnicasian | 0 | 0.97 | 0.12 | 0.984 | Amputation |
| F | 1 | ethnicother | 1.36 (0.87, 2.13) | 0.175 | 1.35 (0.61, 2.96) | 0.452 | Amputation |
| F | 1 | I(egfr/10) | 0.85 (0.75, 0.96) | 0.01 | 0.91 (0.82, 1.01) | 0.067 | Amputation |
| F | 1 | I(hba1c/10) | 1.26 (1.14, 1.38) | <0.001 | 1.24 (1.16, 1.33) | <0.001 | Amputation |
| F | 1 | cv_disease | 2.82 (1.81, 4.37) | <0.001 | 2.51 (1.75, 3.59) | <0.001 | Amputation |
| F | 1 | bmi | 0.97 (0.94, 1) | 0.024 | 0.98 (0.95, 1) | 0.045 | Amputation |
| F | 2 | (Intercept) | -10.16 (-14.02, -6.29) | NA | -9.01 (-12.05, -5.97) | NA | Amputation |
| F | 2 | agenow10 | 1.34 (0.8, 2.25) | 0.266 | 1.45 (0.97, 2.16) | 0.07 | Amputation |
| F | 2 | agedx10 | 0.65 (0.38, 1.12) | 0.123 | 0.6 (0.39, 0.91) | 0.017 | Amputation |
| F | 2 | simd_b | 0.78 (0.51, 1.22) | 0.277 | 0.99 (0.7, 1.39) | 0.937 | Amputation |
| F | 2 | current_smoker | 1.8 (1.13, 2.88) | 0.014 | 1.85 (1.21, 2.82) | 0.004 | Amputation |
| F | 2 | total_chol | 0.98 (0.78, 1.21) | 0.825 | 0.84 (0.71, 1.01) | 0.059 | Amputation |
| F | 2 | hdl | 0.78 (0.39, 1.6) | 0.503 | 0.97 (0.68, 1.4) | 0.887 | Amputation |
| F | 2 | I(sbp/10) | 1.32 (1.14, 1.53) | <0.001 | 1.26 (1.12, 1.41) | <0.001 | Amputation |
| F | 2 | ethnicasian | 0.9 (0.12, 6.62) | 0.915 | 0.7 (0.09, 5.53) | 0.729 | Amputation |
| F | 2 | ethnicother | 1.27 (0.77, 2.09) | 0.351 | 0.47 (0.12, 1.87) | 0.28 | Amputation |
| F | 2 | I(egfr/10) | 0.9 (0.77, 1.04) | 0.148 | 0.93 (0.83, 1.06) | 0.276 | Amputation |
| F | 2 | I(hba1c/10) | 1.32 (1.18, 1.49) | <0.001 | 1.3 (1.19, 1.42) | <0.001 | Amputation |
| F | 2 | cv_disease | 2.04 (1.12, 3.7) | 0.019 | 2.11 (1.32, 3.37) | 0.002 | Amputation |
| F | 2 | bmi | 0.96 (0.93, 1) | 0.028 | 0.96 (0.93, 0.99) | 0.004 | Amputation |
| F | 3 | (Intercept) | -12.47 (-16.81, -8.12) | NA | -11.31 (-14.49, -8.13) | NA | Amputation |
| F | 3 | agenow10 | 1.11 (0.61, 2.03) | 0.739 | 1.22 (0.81, 1.84) | 0.333 | Amputation |
| F | 3 | agedx10 | 1.35 (0.72, 2.54) | 0.345 | 0.92 (0.61, 1.41) | 0.715 | Amputation |
| F | 3 | simd_b | 0.67 (0.41, 1.08) | 0.1 | 0.75 (0.53, 1.06) | 0.104 | Amputation |
| F | 3 | current_smoker | 2.2 (1.29, 3.75) | 0.004 | 2.12 (1.27, 3.54) | 0.005 | Amputation |
| F | 3 | total_chol | 0.87 (0.67, 1.12) | 0.271 | 0.89 (0.74, 1.08) | 0.248 | Amputation |
| F | 3 | hdl | 0.44 (0.19, 1.03) | 0.058 | 1.15 (0.82, 1.63) | 0.413 | Amputation |
| F | 3 | I(sbp/10) | 1.22 (1.03, 1.45) | 0.021 | 1.27 (1.13, 1.42) | <0.001 | Amputation |
| F | 3 | ethnicasian | 0 | 0.971 | 0.47 (0.09, 2.36) | 0.354 | Amputation |
| F | 3 | ethnicother | 1.63 (0.99, 2.68) | 0.054 | 0.88 (0.35, 2.27) | 0.796 | Amputation |
| F | 3 | I(egfr/10) | 1.02 (0.87, 1.2) | 0.817 | 0.96 (0.85, 1.08) | 0.464 | Amputation |
| F | 3 | I(hba1c/10) | 1.21 (1.05, 1.4) | 0.01 | 1.18 (1.07, 1.31) | 0.001 | Amputation |
| F | 3 | cv_disease | 1.85 (0.96, 3.55) | 0.064 | 2.21 (1.39, 3.52) | <0.001 | Amputation |
| F | 3 | bmi | 0.99 (0.95, 1.03) | 0.555 | 0.98 (0.95, 1.01) | 0.205 | Amputation |
| F | 4 | (Intercept) | -10.37 (-14.93, -5.81) | NA | -10.11 (-13.92, -6.3) | NA | Amputation |
| F | 4 | agenow10 | 1.34 (0.73, 2.47) | 0.349 | 1.39 (0.84, 2.29) | 0.202 | Amputation |
| F | 4 | agedx10 | 0.78 (0.41, 1.46) | 0.43 | 0.84 (0.5, 1.41) | 0.508 | Amputation |
| F | 4 | simd_b | 0.78 (0.47, 1.28) | 0.323 | 0.78 (0.51, 1.18) | 0.235 | Amputation |
| F | 4 | current_smoker | 2.89 (1.68, 4.98) | <0.001 | 2.61 (1.59, 4.27) | <0.001 | Amputation |
| F | 4 | total_chol | 0.93 (0.72, 1.21) | 0.604 | 1.02 (0.83, 1.25) | 0.884 | Amputation |
| F | 4 | hdl | 2.03 (1.04, 3.96) | 0.039 | 1.1 (0.72, 1.69) | 0.651 | Amputation |
| F | 4 | I(sbp/10) | 1.12 (0.93, 1.34) | 0.245 | 1.09 (0.94, 1.27) | 0.258 | Amputation |
| F | 4 | ethnicasian | 0 | 0.979 | 0.64 (0.11, 3.59) | 0.607 | Amputation |
| F | 4 | ethnicother | 1 (0.56, 1.79) | 0.999 | 0.65 (0.17, 2.45) | 0.519 | Amputation |
| F | 4 | I(egfr/10) | 1.02 (0.86, 1.21) | 0.845 | 1.06 (0.92, 1.23) | 0.402 | Amputation |
| F | 4 | I(hba1c/10) | 1.24 (1.07, 1.45) | 0.004 | 1.18 (1.04, 1.33) | 0.009 | Amputation |
| F | 4 | cv_disease | 3.64 (1.94, 6.84) | <0.001 | 2.52 (1.41, 4.52) | 0.002 | Amputation |
| F | 4 | bmi | 0.96 (0.91, 1) | 0.051 | 0.95 (0.91, 0.99) | 0.008 | Amputation |
| F | 5 | (Intercept) | -12.21 (-17.34, -7.09) | NA | -10.09 (-14.15, -6.04) | NA | Amputation |
| F | 5 | agenow10 | 1.49 (0.78, 2.84) | 0.224 | 1.45 (0.87, 2.42) | 0.152 | Amputation |
| F | 5 | agedx10 | 0.84 (0.43, 1.62) | 0.602 | 0.82 (0.49, 1.38) | 0.454 | Amputation |
| F | 5 | simd_b | 1.58 (0.92, 2.71) | 0.097 | 1.19 (0.78, 1.82) | 0.425 | Amputation |
| F | 5 | current_smoker | 1.17 (0.52, 2.62) | 0.703 | 1.44 (0.78, 2.65) | 0.243 | Amputation |
| F | 5 | total_chol | 1.24 (0.96, 1.62) | 0.104 | 1.2 (0.98, 1.47) | 0.07 | Amputation |
| F | 5 | hdl | 0.91 (0.41, 2.02) | 0.823 | 1.03 (0.65, 1.62) | 0.893 | Amputation |
| F | 5 | I(sbp/10) | 1.21 (1, 1.47) | 0.053 | 1.09 (0.93, 1.27) | 0.304 | Amputation |
| F | 5 | ethnicasian | 0 | 0.975 | 0 | 0.981 | Amputation |
| F | 5 | ethnicother | 0.34 (0.15, 0.8) | 0.013 | 0.33 (0.06, 1.94) | 0.22 | Amputation |
| F | 5 | I(egfr/10) | 0.88 (0.73, 1.06) | 0.166 | 0.84 (0.72, 0.98) | 0.032 | Amputation |
| F | 5 | I(hba1c/10) | 1.3 (1.12, 1.51) | <0.001 | 1.27 (1.13, 1.42) | <0.001 | Amputation |
| F | 5 | cv_disease | 3.55 (1.79, 7.04) | <0.001 | 2.87 (1.61, 5.12) | <0.001 | Amputation |
| F | 5 | bmi | 0.95 (0.91, 1) | 0.076 | 0.96 (0.92, 1) | 0.072 | Amputation |
| M | 1 | (Intercept) | -4.18 (-4.55, -3.8) | NA | -4.05 (-4.38, -3.73) | NA | Coronary Heart Disease |
| M | 1 | agenow10 | 1.27 (1.21, 1.34) | <0.001 | 1.34 (1.29, 1.4) | <0.001 | Coronary Heart Disease |
| M | 1 | agedx10 | 1.05 (1, 1.11) | 0.062 | 0.99 (0.94, 1.03) | 0.5 | Coronary Heart Disease |
| M | 1 | simd_b | 0.93 (0.89, 0.97) | <0.001 | 0.95 (0.92, 0.98) | 0.003 | Coronary Heart Disease |
| M | 1 | current_smoker | 1.36 (1.3, 1.42) | <0.001 | 1.36 (1.3, 1.43) | <0.001 | Coronary Heart Disease |
| M | 1 | total_chol | 0.99 (0.97, 1.01) | 0.457 | 0.99 (0.97, 1.01) | 0.33 | Coronary Heart Disease |
| M | 1 | hdl | 0.94 (0.87, 1.01) | 0.075 | 1 (0.96, 1.04) | 0.948 | Coronary Heart Disease |
| M | 1 | I(sbp/10) | 0.94 (0.93, 0.96) | <0.001 | 0.95 (0.94, 0.96) | <0.001 | Coronary Heart Disease |
| M | 1 | ethnicasian | 1.04 (0.89, 1.21) | 0.662 | 1.08 (0.92, 1.27) | 0.313 | Coronary Heart Disease |
| M | 1 | ethnicother | 1.19 (1.13, 1.26) | <0.001 | 1.1 (0.94, 1.28) | 0.234 | Coronary Heart Disease |
| M | 1 | I(egfr/10) | 0.91 (0.9, 0.93) | <0.001 | 0.91 (0.9, 0.92) | <0.001 | Coronary Heart Disease |
| M | 1 | I(hba1c/10) | 1.02 (1, 1.03) | 0.017 | 1.01 (1, 1.02) | 0.069 | Coronary Heart Disease |
| M | 1 | cv_disease | 4.84 (4.64, 5.06) | <0.001 | 4.8 (4.63, 4.98) | <0.001 | Coronary Heart Disease |
| M | 1 | bmi | 1.01 (1.01, 1.02) | <0.001 | 1.01 (1.01, 1.01) | <0.001 | Coronary Heart Disease |
| M | 2 | (Intercept) | -4.38 (-4.77, -3.99) | NA | -4.18 (-4.51, -3.86) | NA | Coronary Heart Disease |
| M | 2 | agenow10 | 1.37 (1.31, 1.45) | <0.001 | 1.37 (1.31, 1.43) | <0.001 | Coronary Heart Disease |
| M | 2 | agedx10 | 1 (0.94, 1.05) | 0.927 | 0.99 (0.95, 1.04) | 0.796 | Coronary Heart Disease |
| M | 2 | simd_b | 0.97 (0.93, 1.01) | 0.121 | 0.97 (0.93, 1) | 0.049 | Coronary Heart Disease |
| M | 2 | current_smoker | 1.28 (1.22, 1.35) | <0.001 | 1.27 (1.21, 1.34) | <0.001 | Coronary Heart Disease |
| M | 2 | total_chol | 1.01 (0.99, 1.03) | 0.461 | 1 (0.98, 1.02) | 0.889 | Coronary Heart Disease |
| M | 2 | hdl | 0.88 (0.81, 0.96) | 0.002 | 0.92 (0.88, 0.96) | <0.001 | Coronary Heart Disease |
| M | 2 | I(sbp/10) | 0.95 (0.93, 0.96) | <0.001 | 0.94 (0.93, 0.95) | <0.001 | Coronary Heart Disease |
| M | 2 | ethnicasian | 0.89 (0.74, 1.07) | 0.227 | 0.98 (0.84, 1.16) | 0.825 | Coronary Heart Disease |
| M | 2 | ethnicother | 1.09 (1.03, 1.14) | 0.001 | 1.11 (1, 1.23) | 0.057 | Coronary Heart Disease |
| M | 2 | I(egfr/10) | 0.92 (0.91, 0.94) | <0.001 | 0.92 (0.91, 0.94) | <0.001 | Coronary Heart Disease |
| M | 2 | I(hba1c/10) | 1 (0.99, 1.02) | 0.844 | 1 (0.99, 1.02) | 0.688 | Coronary Heart Disease |
| M | 2 | cv_disease | 4.99 (4.77, 5.22) | <0.001 | 4.92 (4.74, 5.11) | <0.001 | Coronary Heart Disease |
| M | 2 | bmi | 1.01 (1.01, 1.01) | <0.001 | 1.01 (1.01, 1.01) | <0.001 | Coronary Heart Disease |
| M | 3 | (Intercept) | -4.29 (-4.71, -3.87) | NA | -4.5 (-4.85, -4.16) | NA | Coronary Heart Disease |
| M | 3 | agenow10 | 1.45 (1.37, 1.53) | <0.001 | 1.47 (1.4, 1.54) | <0.001 | Coronary Heart Disease |
| M | 3 | agedx10 | 0.98 (0.93, 1.04) | 0.574 | 0.96 (0.91, 1) | 0.064 | Coronary Heart Disease |
| M | 3 | simd_b | 1.04 (0.99, 1.08) | 0.118 | 1.01 (0.97, 1.05) | 0.64 | Coronary Heart Disease |
| M | 3 | current_smoker | 1.34 (1.26, 1.41) | <0.001 | 1.31 (1.24, 1.38) | <0.001 | Coronary Heart Disease |
| M | 3 | total_chol | 0.97 (0.95, 1) | 0.042 | 0.96 (0.94, 0.98) | <0.001 | Coronary Heart Disease |
| M | 3 | hdl | 0.9 (0.82, 0.98) | 0.011 | 0.96 (0.92, 1) | 0.045 | Coronary Heart Disease |
| M | 3 | I(sbp/10) | 0.93 (0.91, 0.95) | <0.001 | 0.94 (0.93, 0.96) | <0.001 | Coronary Heart Disease |
| M | 3 | ethnicasian | 1.1 (0.92, 1.32) | 0.304 | 1 (0.85, 1.17) | 0.969 | Coronary Heart Disease |
| M | 3 | ethnicother | 1.05 (1, 1.11) | 0.044 | 1.14 (1.04, 1.25) | 0.006 | Coronary Heart Disease |
| M | 3 | I(egfr/10) | 0.89 (0.88, 0.9) | <0.001 | 0.9 (0.89, 0.91) | <0.001 | Coronary Heart Disease |
| M | 3 | I(hba1c/10) | 1.02 (1, 1.03) | 0.048 | 1.02 (1, 1.03) | 0.015 | Coronary Heart Disease |
| M | 3 | cv_disease | 4.61 (4.39, 4.84) | <0.001 | 4.58 (4.4, 4.77) | <0.001 | Coronary Heart Disease |
| M | 3 | bmi | 1.02 (1.01, 1.02) | <0.001 | 1.02 (1.01, 1.02) | <0.001 | Coronary Heart Disease |
| M | 4 | (Intercept) | -4.43 (-4.88, -3.97) | NA | -4.44 (-4.85, -4.04) | NA | Coronary Heart Disease |
| M | 4 | agenow10 | 1.45 (1.37, 1.54) | <0.001 | 1.51 (1.44, 1.58) | <0.001 | Coronary Heart Disease |
| M | 4 | agedx10 | 0.97 (0.91, 1.03) | 0.264 | 0.92 (0.88, 0.97) | 0.001 | Coronary Heart Disease |
| M | 4 | simd_b | 0.94 (0.9, 0.99) | 0.011 | 0.94 (0.9, 0.97) | <0.001 | Coronary Heart Disease |
| M | 4 | current_smoker | 1.34 (1.26, 1.43) | <0.001 | 1.38 (1.31, 1.46) | <0.001 | Coronary Heart Disease |
| M | 4 | total_chol | 0.97 (0.95, 1) | 0.048 | 0.97 (0.95, 0.99) | 0.007 | Coronary Heart Disease |
| M | 4 | hdl | 0.8 (0.73, 0.88) | <0.001 | 0.93 (0.88, 0.97) | 0.002 | Coronary Heart Disease |
| M | 4 | I(sbp/10) | 0.95 (0.93, 0.96) | <0.001 | 0.94 (0.93, 0.96) | <0.001 | Coronary Heart Disease |
| M | 4 | ethnicasian | 1.21 (1.01, 1.45) | 0.042 | 1.05 (0.88, 1.26) | 0.588 | Coronary Heart Disease |
| M | 4 | ethnicother | 1.04 (0.98, 1.1) | 0.179 | 1.13 (1.03, 1.24) | 0.009 | Coronary Heart Disease |
| M | 4 | I(egfr/10) | 0.91 (0.89, 0.93) | <0.001 | 0.9 (0.88, 0.91) | <0.001 | Coronary Heart Disease |
| M | 4 | I(hba1c/10) | 1.03 (1.02, 1.05) | <0.001 | 1.03 (1.01, 1.04) | <0.001 | Coronary Heart Disease |
| M | 4 | cv_disease | 4.76 (4.51, 5.02) | <0.001 | 4.87 (4.66, 5.1) | <0.001 | Coronary Heart Disease |
| M | 4 | bmi | 1.01 (1.01, 1.02) | <0.001 | 1.01 (1.01, 1.02) | <0.001 | Coronary Heart Disease |
| M | 5 | (Intercept) | -4.61 (-5.14, -4.08) | NA | -4.29 (-4.73, -3.84) | NA | Coronary Heart Disease |
| M | 5 | agenow10 | 1.49 (1.4, 1.6) | <0.001 | 1.48 (1.4, 1.57) | <0.001 | Coronary Heart Disease |
| M | 5 | agedx10 | 0.98 (0.91, 1.05) | 0.558 | 0.99 (0.93, 1.05) | 0.652 | Coronary Heart Disease |
| M | 5 | simd_b | 0.9 (0.85, 0.95) | <0.001 | 0.91 (0.87, 0.96) | <0.001 | Coronary Heart Disease |
| M | 5 | current_smoker | 1.27 (1.17, 1.38) | <0.001 | 1.28 (1.19, 1.37) | <0.001 | Coronary Heart Disease |
| M | 5 | total_chol | 0.97 (0.94, 1) | 0.035 | 0.94 (0.91, 0.96) | <0.001 | Coronary Heart Disease |
| M | 5 | hdl | 0.88 (0.79, 0.97) | 0.015 | 0.85 (0.8, 0.9) | <0.001 | Coronary Heart Disease |
| M | 5 | I(sbp/10) | 0.93 (0.91, 0.96) | <0.001 | 0.93 (0.92, 0.95) | <0.001 | Coronary Heart Disease |
| M | 5 | ethnicasian | 1.33 (1.15, 1.55) | <0.001 | 1.26 (1.11, 1.42) | <0.001 | Coronary Heart Disease |
| M | 5 | ethnicother | 1.17 (1.1, 1.24) | <0.001 | 1.08 (0.94, 1.25) | 0.263 | Coronary Heart Disease |
| M | 5 | I(egfr/10) | 0.9 (0.88, 0.92) | <0.001 | 0.89 (0.88, 0.91) | <0.001 | Coronary Heart Disease |
| M | 5 | I(hba1c/10) | 1 (0.98, 1.02) | 0.769 | 1.02 (1, 1.03) | 0.092 | Coronary Heart Disease |
| M | 5 | cv_disease | 4.95 (4.66, 5.26) | <0.001 | 4.81 (4.57, 5.07) | <0.001 | Coronary Heart Disease |
| M | 5 | bmi | 1.01 (1.01, 1.02) | <0.001 | 1.01 (1.01, 1.02) | <0.001 | Coronary Heart Disease |
| F | 1 | (Intercept) | -4.5 (-4.94, -4.06) | NA | -4.32 (-4.68, -3.97) | NA | Coronary Heart Disease |
| F | 1 | agenow10 | 1.42 (1.34, 1.51) | <0.001 | 1.39 (1.32, 1.46) | <0.001 | Coronary Heart Disease |
| F | 1 | agedx10 | 0.94 (0.88, 1) | 0.051 | 0.94 (0.9, 0.99) | 0.02 | Coronary Heart Disease |
| F | 1 | simd_b | 0.94 (0.9, 0.99) | 0.021 | 0.96 (0.93, 1) | 0.062 | Coronary Heart Disease |
| F | 1 | current_smoker | 1.37 (1.3, 1.45) | <0.001 | 1.43 (1.36, 1.51) | <0.001 | Coronary Heart Disease |
| F | 1 | total_chol | 0.99 (0.97, 1.02) | 0.472 | 0.96 (0.94, 0.98) | <0.001 | Coronary Heart Disease |
| F | 1 | hdl | 0.81 (0.75, 0.88) | <0.001 | 0.96 (0.92, 1) | 0.049 | Coronary Heart Disease |
| F | 1 | I(sbp/10) | 0.96 (0.94, 0.98) | <0.001 | 0.96 (0.95, 0.98) | <0.001 | Coronary Heart Disease |
| F | 1 | ethnicasian | 0.83 (0.62, 1.11) | 0.209 | 0.92 (0.75, 1.12) | 0.384 | Coronary Heart Disease |
| F | 1 | ethnicother | 1.12 (1.06, 1.2) | <0.001 | 1.08 (0.94, 1.24) | 0.265 | Coronary Heart Disease |
| F | 1 | I(egfr/10) | 0.94 (0.93, 0.96) | <0.001 | 0.92 (0.91, 0.93) | <0.001 | Coronary Heart Disease |
| F | 1 | I(hba1c/10) | 0.99 (0.98, 1.01) | 0.466 | 1.01 (0.99, 1.02) | 0.32 | Coronary Heart Disease |
| F | 1 | cv_disease | 6.27 (5.95, 6.6) | <0.001 | 6.35 (6.08, 6.63) | <0.001 | Coronary Heart Disease |
| F | 1 | bmi | 1.01 (1, 1.01) | <0.001 | 1.01 (1, 1.01) | 0.002 | Coronary Heart Disease |
| F | 2 | (Intercept) | -5.08 (-5.55, -4.6) | NA | -5.02 (-5.43, -4.61) | NA | Coronary Heart Disease |
| F | 2 | agenow10 | 1.51 (1.42, 1.62) | <0.001 | 1.47 (1.39, 1.55) | <0.001 | Coronary Heart Disease |
| F | 2 | agedx10 | 0.93 (0.87, 0.99) | 0.029 | 0.95 (0.9, 1.01) | 0.077 | Coronary Heart Disease |
| F | 2 | simd_b | 0.95 (0.91, 1) | 0.075 | 0.96 (0.92, 1) | 0.052 | Coronary Heart Disease |
| F | 2 | current_smoker | 1.5 (1.41, 1.59) | <0.001 | 1.48 (1.4, 1.56) | <0.001 | Coronary Heart Disease |
| F | 2 | total_chol | 0.98 (0.95, 1) | 0.086 | 0.97 (0.95, 0.99) | 0.007 | Coronary Heart Disease |
| F | 2 | hdl | 0.88 (0.81, 0.96) | 0.003 | 0.94 (0.9, 0.98) | 0.009 | Coronary Heart Disease |
| F | 2 | I(sbp/10) | 0.96 (0.95, 0.98) | <0.001 | 0.96 (0.95, 0.98) | <0.001 | Coronary Heart Disease |
| F | 2 | ethnicasian | 0.75 (0.55, 1.03) | 0.08 | 0.91 (0.64, 1.29) | 0.569 | Coronary Heart Disease |
| F | 2 | ethnicother | 1.15 (1.08, 1.22) | <0.001 | 1.06 (0.92, 1.22) | 0.439 | Coronary Heart Disease |
| F | 2 | I(egfr/10) | 0.93 (0.91, 0.95) | <0.001 | 0.92 (0.91, 0.93) | <0.001 | Coronary Heart Disease |
| F | 2 | I(hba1c/10) | 1.01 (0.99, 1.03) | 0.238 | 1.02 (1, 1.04) | 0.02 | Coronary Heart Disease |
| F | 2 | cv_disease | 6.65 (6.29, 7.04) | <0.001 | 6.52 (6.22, 6.83) | <0.001 | Coronary Heart Disease |
| F | 2 | bmi | 1.01 (1, 1.01) | 0.016 | 1.01 (1, 1.01) | 0.006 | Coronary Heart Disease |
| F | 3 | (Intercept) | -4.75 (-5.31, -4.19) | NA | -4.68 (-5.13, -4.23) | NA | Coronary Heart Disease |
| F | 3 | agenow10 | 1.49 (1.38, 1.6) | <0.001 | 1.47 (1.39, 1.56) | <0.001 | Coronary Heart Disease |
| F | 3 | agedx10 | 0.94 (0.87, 1.02) | 0.121 | 0.91 (0.85, 0.96) | 0.002 | Coronary Heart Disease |
| F | 3 | simd_b | 0.97 (0.91, 1.03) | 0.313 | 0.97 (0.92, 1.02) | 0.174 | Coronary Heart Disease |
| F | 3 | current_smoker | 1.36 (1.25, 1.47) | <0.001 | 1.4 (1.3, 1.5) | <0.001 | Coronary Heart Disease |
| F | 3 | total_chol | 0.95 (0.92, 0.99) | 0.005 | 0.96 (0.93, 0.99) | 0.003 | Coronary Heart Disease |
| F | 3 | hdl | 0.77 (0.69, 0.84) | <0.001 | 0.9 (0.85, 0.95) | <0.001 | Coronary Heart Disease |
| F | 3 | I(sbp/10) | 0.98 (0.96, 1) | 0.115 | 0.99 (0.97, 1.01) | 0.154 | Coronary Heart Disease |
| F | 3 | ethnicasian | 1.17 (0.87, 1.58) | 0.293 | 0.99 (0.8, 1.22) | 0.926 | Coronary Heart Disease |
| F | 3 | ethnicother | 1.07 (1, 1.15) | 0.055 | 1 (0.87, 1.15) | 0.976 | Coronary Heart Disease |
| F | 3 | I(egfr/10) | 0.89 (0.87, 0.91) | <0.001 | 0.88 (0.86, 0.89) | <0.001 | Coronary Heart Disease |
| F | 3 | I(hba1c/10) | 1.02 (0.99, 1.04) | 0.132 | 1.04 (1.02, 1.06) | <0.001 | Coronary Heart Disease |
| F | 3 | cv_disease | 6.33 (5.92, 6.77) | <0.001 | 6.46 (6.12, 6.82) | <0.001 | Coronary Heart Disease |
| F | 3 | bmi | 1 (1, 1.01) | 0.096 | 1 (1, 1.01) | 0.093 | Coronary Heart Disease |
| F | 4 | (Intercept) | -5.55 (-6.18, -4.92) | NA | -5.32 (-5.86, -4.78) | NA | Coronary Heart Disease |
| F | 4 | agenow10 | 1.3 (1.2, 1.42) | <0.001 | 1.38 (1.29, 1.48) | <0.001 | Coronary Heart Disease |
| F | 4 | agedx10 | 1.15 (1.05, 1.25) | 0.002 | 1.04 (0.97, 1.11) | 0.319 | Coronary Heart Disease |
| F | 4 | simd_b | 0.9 (0.84, 0.96) | 0.001 | 0.92 (0.87, 0.97) | 0.004 | Coronary Heart Disease |
| F | 4 | current_smoker | 1.41 (1.29, 1.55) | <0.001 | 1.39 (1.28, 1.51) | <0.001 | Coronary Heart Disease |
| F | 4 | total_chol | 0.93 (0.9, 0.97) | <0.001 | 0.95 (0.92, 0.98) | 0.004 | Coronary Heart Disease |
| F | 4 | hdl | 0.86 (0.77, 0.96) | 0.007 | 0.93 (0.87, 0.99) | 0.015 | Coronary Heart Disease |
| F | 4 | I(sbp/10) | 0.97 (0.95, 1) | 0.026 | 0.98 (0.96, 1) | 0.061 | Coronary Heart Disease |
| F | 4 | ethnicasian | 1.35 (1.05, 1.73) | 0.02 | 1.33 (1.08, 1.65) | 0.01 | Coronary Heart Disease |
| F | 4 | ethnicother | 1.03 (0.95, 1.11) | 0.541 | 1 (0.86, 1.16) | 0.983 | Coronary Heart Disease |
| F | 4 | I(egfr/10) | 0.89 (0.87, 0.91) | <0.001 | 0.86 (0.85, 0.88) | <0.001 | Coronary Heart Disease |
| F | 4 | I(hba1c/10) | 1.05 (1.02, 1.08) | <0.001 | 1.04 (1.02, 1.07) | 0.001 | Coronary Heart Disease |
| F | 4 | cv_disease | 6.58 (6.1, 7.09) | <0.001 | 6.39 (6.01, 6.8) | <0.001 | Coronary Heart Disease |
| F | 4 | bmi | 1.01 (1.01, 1.02) | <0.001 | 1.01 (1.01, 1.02) | <0.001 | Coronary Heart Disease |
| F | 5 | (Intercept) | -6.25 (-6.99, -5.5) | NA | -5.78 (-6.42, -5.15) | NA | Coronary Heart Disease |
| F | 5 | agenow10 | 1.59 (1.44, 1.75) | <0.001 | 1.6 (1.48, 1.74) | <0.001 | Coronary Heart Disease |
| F | 5 | agedx10 | 0.96 (0.87, 1.06) | 0.376 | 0.93 (0.86, 1.01) | 0.094 | Coronary Heart Disease |
| F | 5 | simd_b | 0.93 (0.86, 1.01) | 0.083 | 0.93 (0.87, 0.99) | 0.026 | Coronary Heart Disease |
| F | 5 | current_smoker | 1.48 (1.32, 1.67) | <0.001 | 1.41 (1.27, 1.57) | <0.001 | Coronary Heart Disease |
| F | 5 | total_chol | 0.95 (0.91, 0.99) | 0.026 | 0.92 (0.89, 0.96) | <0.001 | Coronary Heart Disease |
| F | 5 | hdl | 0.83 (0.73, 0.94) | 0.003 | 0.91 (0.85, 0.99) | 0.023 | Coronary Heart Disease |
| F | 5 | I(sbp/10) | 0.98 (0.95, 1.01) | 0.207 | 0.97 (0.95, 0.99) | 0.017 | Coronary Heart Disease |
| F | 5 | ethnicasian | 1.13 (0.89, 1.43) | 0.314 | 1.23 (0.98, 1.54) | 0.076 | Coronary Heart Disease |
| F | 5 | ethnicother | 1.08 (0.99, 1.18) | 0.101 | 1.1 (0.89, 1.35) | 0.382 | Coronary Heart Disease |
| F | 5 | I(egfr/10) | 0.9 (0.87, 0.92) | <0.001 | 0.9 (0.88, 0.92) | <0.001 | Coronary Heart Disease |
| F | 5 | I(hba1c/10) | 1.05 (1.02, 1.08) | <0.001 | 1.04 (1.01, 1.06) | 0.008 | Coronary Heart Disease |
| F | 5 | cv_disease | 6.17 (5.64, 6.74) | <0.001 | 6.46 (6, 6.97) | <0.001 | Coronary Heart Disease |
| F | 5 | bmi | 1.02 (1.01, 1.02) | <0.001 | 1.02 (1.01, 1.02) | <0.001 | Coronary Heart Disease |
| M | 1 | (Intercept) | -7.72 (-8.41, -7.02) | NA | -7.7 (-8.29, -7.11) | NA | Heart Failure |
| M | 1 | agenow10 | 2.02 (1.85, 2.21) | <0.001 | 2.02 (1.88, 2.17) | <0.001 | Heart Failure |
| M | 1 | agedx10 | 0.9 (0.82, 0.99) | 0.03 | 0.89 (0.83, 0.96) | 0.003 | Heart Failure |
| M | 1 | simd_b | 0.88 (0.82, 0.95) | <0.001 | 0.91 (0.86, 0.96) | 0.002 | Heart Failure |
| M | 1 | current_smoker | 1.49 (1.37, 1.62) | <0.001 | 1.53 (1.42, 1.64) | <0.001 | Heart Failure |
| M | 1 | total_chol | 0.95 (0.91, 0.99) | 0.022 | 0.96 (0.93, 1) | 0.025 | Heart Failure |
| M | 1 | hdl | 1.19 (1.05, 1.35) | 0.007 | 1.13 (1.06, 1.2) | <0.001 | Heart Failure |
| M | 1 | I(sbp/10) | 0.91 (0.88, 0.93) | <0.001 | 0.92 (0.9, 0.94) | <0.001 | Heart Failure |
| M | 1 | ethnicasian | 0.65 (0.43, 0.96) | 0.032 | 0.99 (0.77, 1.26) | 0.923 | Heart Failure |
| M | 1 | ethnicother | 1.03 (0.93, 1.13) | 0.581 | 0.95 (0.77, 1.18) | 0.638 | Heart Failure |
| M | 1 | I(egfr/10) | 0.84 (0.82, 0.87) | <0.001 | 0.84 (0.82, 0.85) | <0.001 | Heart Failure |
| M | 1 | I(hba1c/10) | 1.08 (1.05, 1.1) | <0.001 | 1.08 (1.06, 1.1) | <0.001 | Heart Failure |
| M | 1 | cv_disease | 2.5 (2.3, 2.71) | <0.001 | 2.49 (2.33, 2.66) | <0.001 | Heart Failure |
| M | 1 | bmi | 1.04 (1.04, 1.05) | <0.001 | 1.04 (1.03, 1.05) | <0.001 | Heart Failure |
| M | 2 | (Intercept) | -9.07 (-9.79, -8.36) | NA | -8.29 (-8.88, -7.7) | NA | Heart Failure |
| M | 2 | agenow10 | 2.24 (2.05, 2.46) | <0.001 | 2.04 (1.9, 2.19) | <0.001 | Heart Failure |
| M | 2 | agedx10 | 0.85 (0.78, 0.94) | <0.001 | 0.9 (0.83, 0.97) | 0.004 | Heart Failure |
| M | 2 | simd_b | 1.01 (0.94, 1.09) | 0.723 | 1 (0.94, 1.07) | 0.916 | Heart Failure |
| M | 2 | current_smoker | 1.39 (1.27, 1.52) | <0.001 | 1.32 (1.21, 1.43) | <0.001 | Heart Failure |
| M | 2 | total_chol | 0.97 (0.93, 1.02) | 0.208 | 0.97 (0.94, 1.01) | 0.149 | Heart Failure |
| M | 2 | hdl | 1.17 (1.01, 1.34) | 0.031 | 1.09 (1.02, 1.17) | 0.017 | Heart Failure |
| M | 2 | I(sbp/10) | 0.94 (0.91, 0.97) | <0.001 | 0.93 (0.91, 0.95) | <0.001 | Heart Failure |
| M | 2 | ethnicasian | 0.75 (0.48, 1.16) | 0.191 | 0.92 (0.61, 1.39) | 0.69 | Heart Failure |
| M | 2 | ethnicother | 1.12 (1.03, 1.23) | 0.01 | 0.91 (0.74, 1.11) | 0.334 | Heart Failure |
| M | 2 | I(egfr/10) | 0.85 (0.82, 0.87) | <0.001 | 0.84 (0.82, 0.86) | <0.001 | Heart Failure |
| M | 2 | I(hba1c/10) | 1.09 (1.06, 1.12) | <0.001 | 1.1 (1.08, 1.12) | <0.001 | Heart Failure |
| M | 2 | cv_disease | 2.7 (2.49, 2.94) | <0.001 | 2.66 (2.48, 2.85) | <0.001 | Heart Failure |
| M | 2 | bmi | 1.05 (1.04, 1.06) | <0.001 | 1.04 (1.04, 1.05) | <0.001 | Heart Failure |
| M | 3 | (Intercept) | -8.16 (-8.91, -7.41) | NA | -8.51 (-9.14, -7.87) | NA | Heart Failure |
| M | 3 | agenow10 | 2.27 (2.05, 2.5) | <0.001 | 2.16 (2, 2.34) | <0.001 | Heart Failure |
| M | 3 | agedx10 | 0.87 (0.79, 0.96) | 0.006 | 0.9 (0.83, 0.97) | 0.007 | Heart Failure |
| M | 3 | simd_b | 1.07 (0.99, 1.16) | 0.089 | 1.05 (0.99, 1.13) | 0.113 | Heart Failure |
| M | 3 | current_smoker | 1.53 (1.38, 1.69) | <0.001 | 1.48 (1.35, 1.63) | <0.001 | Heart Failure |
| M | 3 | total_chol | 0.96 (0.92, 1.01) | 0.115 | 0.96 (0.92, 0.99) | 0.023 | Heart Failure |
| M | 3 | hdl | 1.17 (1.01, 1.36) | 0.034 | 1.1 (1.02, 1.18) | 0.011 | Heart Failure |
| M | 3 | I(sbp/10) | 0.87 (0.84, 0.9) | <0.001 | 0.9 (0.88, 0.92) | <0.001 | Heart Failure |
| M | 3 | ethnicasian | 0.78 (0.5, 1.22) | 0.283 | 0.98 (0.75, 1.28) | 0.879 | Heart Failure |
| M | 3 | ethnicother | 1.12 (1.03, 1.23) | 0.012 | 1.2 (1, 1.44) | 0.05 | Heart Failure |
| M | 3 | I(egfr/10) | 0.8 (0.78, 0.82) | <0.001 | 0.82 (0.8, 0.84) | <0.001 | Heart Failure |
| M | 3 | I(hba1c/10) | 1.11 (1.08, 1.14) | <0.001 | 1.1 (1.08, 1.13) | <0.001 | Heart Failure |
| M | 3 | cv_disease | 2.49 (2.28, 2.72) | <0.001 | 2.62 (2.44, 2.82) | <0.001 | Heart Failure |
| M | 3 | bmi | 1.06 (1.05, 1.06) | <0.001 | 1.05 (1.05, 1.06) | <0.001 | Heart Failure |
| M | 4 | (Intercept) | -9.24 (-10.07, -8.42) | NA | -8.7 (-9.38, -8.02) | NA | Heart Failure |
| M | 4 | agenow10 | 2.48 (2.23, 2.76) | <0.001 | 2.34 (2.15, 2.55) | <0.001 | Heart Failure |
| M | 4 | agedx10 | 0.8 (0.72, 0.89) | <0.001 | 0.81 (0.75, 0.89) | <0.001 | Heart Failure |
| M | 4 | simd_b | 0.9 (0.83, 0.99) | 0.022 | 0.93 (0.87, 1) | 0.049 | Heart Failure |
| M | 4 | current_smoker | 1.72 (1.53, 1.93) | <0.001 | 1.66 (1.5, 1.83) | <0.001 | Heart Failure |
| M | 4 | total_chol | 0.93 (0.88, 0.98) | 0.008 | 0.92 (0.88, 0.96) | <0.001 | Heart Failure |
| M | 4 | hdl | 1.21 (1.04, 1.42) | 0.016 | 1.15 (1.06, 1.24) | <0.001 | Heart Failure |
| M | 4 | I(sbp/10) | 0.92 (0.89, 0.96) | <0.001 | 0.93 (0.9, 0.95) | <0.001 | Heart Failure |
| M | 4 | ethnicasian | 1.55 (1.07, 2.24) | 0.021 | 1.2 (0.89, 1.62) | 0.221 | Heart Failure |
| M | 4 | ethnicother | 1.08 (0.98, 1.2) | 0.111 | 0.96 (0.78, 1.19) | 0.72 | Heart Failure |
| M | 4 | I(egfr/10) | 0.77 (0.74, 0.79) | <0.001 | 0.78 (0.75, 0.8) | <0.001 | Heart Failure |
| M | 4 | I(hba1c/10) | 1.14 (1.11, 1.18) | <0.001 | 1.13 (1.1, 1.16) | <0.001 | Heart Failure |
| M | 4 | cv_disease | 2.46 (2.23, 2.71) | <0.001 | 2.37 (2.19, 2.58) | <0.001 | Heart Failure |
| M | 4 | bmi | 1.07 (1.06, 1.08) | <0.001 | 1.06 (1.05, 1.07) | <0.001 | Heart Failure |
| M | 5 | (Intercept) | -8.95 (-9.93, -7.96) | NA | -8.52 (-9.32, -7.72) | NA | Heart Failure |
| M | 5 | agenow10 | 2.44 (2.16, 2.75) | <0.001 | 2.19 (1.98, 2.42) | <0.001 | Heart Failure |
| M | 5 | agedx10 | 0.84 (0.75, 0.95) | 0.006 | 0.91 (0.82, 1) | 0.054 | Heart Failure |
| M | 5 | simd_b | 0.94 (0.85, 1.04) | 0.216 | 0.93 (0.85, 1.01) | 0.071 | Heart Failure |
| M | 5 | current_smoker | 1.23 (1.04, 1.44) | 0.015 | 1.28 (1.08, 1.51) | 0.005 | Heart Failure |
| M | 5 | total_chol | 0.99 (0.93, 1.05) | 0.64 | 0.97 (0.93, 1.02) | 0.232 | Heart Failure |
| M | 5 | hdl | 1.11 (0.92, 1.33) | 0.273 | 1.02 (0.93, 1.13) | 0.638 | Heart Failure |
| M | 5 | I(sbp/10) | 0.9 (0.86, 0.94) | <0.001 | 0.92 (0.89, 0.95) | <0.001 | Heart Failure |
| M | 5 | ethnicasian | 1.02 (0.72, 1.43) | 0.92 | 0.98 (0.79, 1.22) | 0.857 | Heart Failure |
| M | 5 | ethnicother | 1.16 (1.04, 1.3) | 0.007 | 0.98 (0.76, 1.27) | 0.869 | Heart Failure |
| M | 5 | I(egfr/10) | 0.8 (0.77, 0.83) | <0.001 | 0.79 (0.77, 0.82) | <0.001 | Heart Failure |
| M | 5 | I(hba1c/10) | 1.1 (1.06, 1.14) | <0.001 | 1.11 (1.08, 1.15) | <0.001 | Heart Failure |
| M | 5 | cv_disease | 2.26 (2.01, 2.54) | <0.001 | 2.31 (2.1, 2.55) | <0.001 | Heart Failure |
| M | 5 | bmi | 1.05 (1.04, 1.06) | <0.001 | 1.05 (1.04, 1.06) | <0.001 | Heart Failure |
| F | 1 | (Intercept) | -7.89 (-8.63, -7.14) | NA | -7.99 (-8.59, -7.38) | NA | Heart Failure |
| F | 1 | agenow10 | 2.08 (1.88, 2.29) | <0.001 | 1.98 (1.83, 2.14) | <0.001 | Heart Failure |
| F | 1 | agedx10 | 0.89 (0.81, 0.99) | 0.026 | 0.9 (0.83, 0.97) | 0.009 | Heart Failure |
| F | 1 | simd_b | 0.95 (0.88, 1.03) | 0.213 | 0.99 (0.93, 1.06) | 0.79 | Heart Failure |
| F | 1 | current_smoker | 1.41 (1.28, 1.55) | <0.001 | 1.47 (1.36, 1.59) | <0.001 | Heart Failure |
| F | 1 | total_chol | 0.86 (0.82, 0.9) | <0.001 | 0.89 (0.85, 0.92) | <0.001 | Heart Failure |
| F | 1 | hdl | 0.89 (0.78, 1.02) | 0.095 | 1.08 (1.01, 1.16) | 0.033 | Heart Failure |
| F | 1 | I(sbp/10) | 0.95 (0.92, 0.98) | 0.001 | 0.96 (0.94, 0.98) | 0.001 | Heart Failure |
| F | 1 | ethnicasian | 1.02 (0.61, 1.72) | 0.933 | 0.93 (0.58, 1.47) | 0.734 | Heart Failure |
| F | 1 | ethnicother | 1.15 (1.04, 1.28) | 0.006 | 1.15 (0.95, 1.4) | 0.16 | Heart Failure |
| F | 1 | I(egfr/10) | 0.83 (0.81, 0.86) | <0.001 | 0.82 (0.8, 0.84) | <0.001 | Heart Failure |
| F | 1 | I(hba1c/10) | 1.1 (1.07, 1.13) | <0.001 | 1.11 (1.09, 1.14) | <0.001 | Heart Failure |
| F | 1 | cv_disease | 2.18 (1.98, 2.41) | <0.001 | 2.41 (2.23, 2.61) | <0.001 | Heart Failure |
| F | 1 | bmi | 1.04 (1.03, 1.05) | <0.001 | 1.04 (1.03, 1.04) | <0.001 | Heart Failure |
| F | 2 | (Intercept) | -9.79 (-10.6, -8.97) | NA | -9.96 (-10.61, -9.3) | NA | Heart Failure |
| F | 2 | agenow10 | 2.34 (2.11, 2.59) | <0.001 | 2.18 (2.01, 2.37) | <0.001 | Heart Failure |
| F | 2 | agedx10 | 0.88 (0.79, 0.97) | 0.015 | 0.96 (0.88, 1.05) | 0.363 | Heart Failure |
| F | 2 | simd_b | 0.98 (0.9, 1.07) | 0.711 | 0.95 (0.89, 1.02) | 0.155 | Heart Failure |
| F | 2 | current_smoker | 1.79 (1.62, 1.98) | <0.001 | 1.74 (1.58, 1.92) | <0.001 | Heart Failure |
| F | 2 | total_chol | 0.96 (0.92, 1.01) | 0.087 | 0.93 (0.9, 0.97) | <0.001 | Heart Failure |
| F | 2 | hdl | 0.8 (0.69, 0.92) | 0.002 | 0.94 (0.87, 1.01) | 0.083 | Heart Failure |
| F | 2 | I(sbp/10) | 0.97 (0.94, 1) | 0.042 | 0.97 (0.94, 0.99) | 0.008 | Heart Failure |
| F | 2 | ethnicasian | 0.88 (0.5, 1.57) | 0.665 | 1.1 (0.7, 1.74) | 0.665 | Heart Failure |
| F | 2 | ethnicother | 1.12 (1.01, 1.24) | 0.026 | 1.16 (0.94, 1.44) | 0.157 | Heart Failure |
| F | 2 | I(egfr/10) | 0.82 (0.79, 0.84) | <0.001 | 0.83 (0.81, 0.85) | <0.001 | Heart Failure |
| F | 2 | I(hba1c/10) | 1.16 (1.13, 1.2) | <0.001 | 1.15 (1.12, 1.18) | <0.001 | Heart Failure |
| F | 2 | cv_disease | 2.7 (2.44, 2.98) | <0.001 | 2.54 (2.34, 2.75) | <0.001 | Heart Failure |
| F | 2 | bmi | 1.04 (1.04, 1.05) | <0.001 | 1.04 (1.04, 1.05) | <0.001 | Heart Failure |
| F | 3 | (Intercept) | -9.82 (-10.75, -8.89) | NA | -9.6 (-10.32, -8.88) | NA | Heart Failure |
| F | 3 | agenow10 | 2.38 (2.11, 2.69) | <0.001 | 2.21 (2.01, 2.42) | <0.001 | Heart Failure |
| F | 3 | agedx10 | 0.94 (0.83, 1.06) | 0.329 | 0.94 (0.86, 1.03) | 0.201 | Heart Failure |
| F | 3 | simd_b | 1.01 (0.92, 1.12) | 0.767 | 0.97 (0.89, 1.04) | 0.388 | Heart Failure |
| F | 3 | current_smoker | 1.53 (1.33, 1.75) | <0.001 | 1.47 (1.31, 1.64) | <0.001 | Heart Failure |
| F | 3 | total_chol | 0.94 (0.89, 1) | 0.04 | 0.93 (0.88, 0.97) | <0.001 | Heart Failure |
| F | 3 | hdl | 0.73 (0.62, 0.85) | <0.001 | 1.01 (0.92, 1.1) | 0.854 | Heart Failure |
| F | 3 | I(sbp/10) | 0.94 (0.91, 0.98) | 0.002 | 0.97 (0.94, 1) | 0.024 | Heart Failure |
| F | 3 | ethnicasian | 1.7 (1.01, 2.87) | 0.044 | 1.09 (0.81, 1.47) | 0.566 | Heart Failure |
| F | 3 | ethnicother | 1.19 (1.07, 1.33) | 0.002 | 1.13 (0.9, 1.42) | 0.281 | Heart Failure |
| F | 3 | I(egfr/10) | 0.83 (0.81, 0.86) | <0.001 | 0.82 (0.8, 0.84) | <0.001 | Heart Failure |
| F | 3 | I(hba1c/10) | 1.09 (1.05, 1.13) | <0.001 | 1.1 (1.07, 1.14) | <0.001 | Heart Failure |
| F | 3 | cv_disease | 2.17 (1.92, 2.45) | <0.001 | 2.27 (2.07, 2.5) | <0.001 | Heart Failure |
| F | 3 | bmi | 1.05 (1.04, 1.06) | <0.001 | 1.04 (1.04, 1.05) | <0.001 | Heart Failure |
| F | 4 | (Intercept) | -8.79 (-9.83, -7.75) | NA | -8.93 (-9.78, -8.07) | NA | Heart Failure |
| F | 4 | agenow10 | 2.14 (1.87, 2.44) | <0.001 | 2.27 (2.04, 2.53) | <0.001 | Heart Failure |
| F | 4 | agedx10 | 0.95 (0.83, 1.09) | 0.504 | 0.88 (0.79, 0.98) | 0.02 | Heart Failure |
| F | 4 | simd_b | 1.05 (0.94, 1.17) | 0.392 | 1.06 (0.97, 1.16) | 0.172 | Heart Failure |
| F | 4 | current_smoker | 1.6 (1.36, 1.87) | <0.001 | 1.55 (1.34, 1.79) | <0.001 | Heart Failure |
| F | 4 | total_chol | 0.9 (0.85, 0.95) | <0.001 | 0.92 (0.88, 0.96) | <0.001 | Heart Failure |
| F | 4 | hdl | 0.95 (0.8, 1.13) | 0.572 | 1 (0.91, 1.1) | 0.951 | Heart Failure |
| F | 4 | I(sbp/10) | 0.92 (0.88, 0.96) | <0.001 | 0.94 (0.9, 0.97) | <0.001 | Heart Failure |
| F | 4 | ethnicasian | 1.66 (1.07, 2.59) | 0.024 | 1.27 (0.87, 1.84) | 0.205 | Heart Failure |
| F | 4 | ethnicother | 1.04 (0.92, 1.18) | 0.539 | 0.98 (0.78, 1.22) | 0.829 | Heart Failure |
| F | 4 | I(egfr/10) | 0.79 (0.76, 0.82) | <0.001 | 0.79 (0.77, 0.82) | <0.001 | Heart Failure |
| F | 4 | I(hba1c/10) | 1.14 (1.1, 1.19) | <0.001 | 1.14 (1.1, 1.17) | <0.001 | Heart Failure |
| F | 4 | cv_disease | 2.47 (2.16, 2.82) | <0.001 | 2.45 (2.19, 2.73) | <0.001 | Heart Failure |
| F | 4 | bmi | 1.04 (1.03, 1.05) | <0.001 | 1.04 (1.03, 1.05) | <0.001 | Heart Failure |
| F | 5 | (Intercept) | -11.09 (-12.34, -9.84) | NA | -10.8 (-11.85, -9.75) | NA | Heart Failure |
| F | 5 | agenow10 | 2.63 (2.25, 3.07) | <0.001 | 2.4 (2.11, 2.73) | <0.001 | Heart Failure |
| F | 5 | agedx10 | 0.92 (0.79, 1.07) | 0.288 | 0.98 (0.86, 1.11) | 0.699 | Heart Failure |
| F | 5 | simd_b | 0.99 (0.87, 1.12) | 0.847 | 1.11 (1, 1.24) | 0.041 | Heart Failure |
| F | 5 | current_smoker | 1.86 (1.54, 2.25) | <0.001 | 1.74 (1.47, 2.06) | <0.001 | Heart Failure |
| F | 5 | total_chol | 1 (0.93, 1.07) | 0.954 | 0.98 (0.92, 1.03) | 0.406 | Heart Failure |
| F | 5 | hdl | 0.86 (0.71, 1.05) | 0.146 | 0.95 (0.85, 1.07) | 0.393 | Heart Failure |
| F | 5 | I(sbp/10) | 0.96 (0.91, 1.01) | 0.081 | 0.96 (0.92, 1) | 0.031 | Heart Failure |
| F | 5 | ethnicasian | 1.4 (0.91, 2.15) | 0.121 | 1.47 (1.05, 2.04) | 0.024 | Heart Failure |
| F | 5 | ethnicother | 1.06 (0.92, 1.22) | 0.452 | 0.88 (0.66, 1.16) | 0.346 | Heart Failure |
| F | 5 | I(egfr/10) | 0.81 (0.77, 0.84) | <0.001 | 0.82 (0.79, 0.85) | <0.001 | Heart Failure |
| F | 5 | I(hba1c/10) | 1.12 (1.07, 1.17) | <0.001 | 1.1 (1.06, 1.14) | <0.001 | Heart Failure |
| F | 5 | cv_disease | 2.73 (2.33, 3.18) | <0.001 | 2.72 (2.39, 3.09) | <0.001 | Heart Failure |
| F | 5 | bmi | 1.04 (1.03, 1.05) | <0.001 | 1.04 (1.03, 1.05) | <0.001 | Heart Failure |
| M | 1 | (Intercept) | -13.93 (-17.3, -10.57) | NA | -14.57 (-17.44, -11.7) | NA | Renal Failure |
| M | 1 | agenow10 | 2.22 (1.32, 3.73) | 0.003 | 2.09 (1.34, 3.24) | 0.001 | Renal Failure |
| M | 1 | agedx10 | 0.5 (0.29, 0.87) | 0.013 | 0.6 (0.38, 0.95) | 0.031 | Renal Failure |
| M | 1 | simd_b | 0.91 (0.58, 1.42) | 0.683 | 0.97 (0.66, 1.43) | 0.887 | Renal Failure |
| M | 1 | current_smoker | 1.16 (0.71, 1.9) | 0.551 | 1.05 (0.67, 1.66) | 0.822 | Renal Failure |
| M | 1 | total_chol | 0.81 (0.63, 1.03) | 0.081 | 0.93 (0.77, 1.13) | 0.46 | Renal Failure |
| M | 1 | hdl | 1.42 (0.7, 2.87) | 0.326 | 1.02 (0.67, 1.55) | 0.924 | Renal Failure |
| M | 1 | I(sbp/10) | 1.37 (1.18, 1.6) | <0.001 | 1.3 (1.14, 1.48) | <0.001 | Renal Failure |
| M | 1 | ethnicasian | 1.42 (0.34, 5.96) | 0.628 | 2.24 (0.84, 6) | 0.106 | Renal Failure |
| M | 1 | ethnicother | 2.15 (1.31, 3.52) | 0.002 | 1.39 (0.53, 3.65) | 0.5 | Renal Failure |
| M | 1 | I(hba1c/10) | 1.26 (1.13, 1.41) | <0.001 | 1.26 (1.14, 1.38) | <0.001 | Renal Failure |
| M | 1 | cv_disease | 1.03 (0.51, 2.04) | 0.942 | 1.28 (0.73, 2.22) | 0.388 | Renal Failure |
| M | 1 | bmi | 0.99 (0.95, 1.03) | 0.498 | 1 (0.97, 1.03) | 0.978 | Renal Failure |
| M | 2 | (Intercept) | -13.75 (-17.48, -10.03) | NA | -11.97 (-14.93, -9) | NA | Renal Failure |
| M | 2 | agenow10 | 2.49 (1.45, 4.27) | <0.001 | 2.11 (1.38, 3.25) | <0.001 | Renal Failure |
| M | 2 | agedx10 | 0.49 (0.28, 0.86) | 0.013 | 0.53 (0.34, 0.84) | 0.006 | Renal Failure |
| M | 2 | simd_b | 1.04 (0.65, 1.65) | 0.883 | 1.07 (0.73, 1.55) | 0.736 | Renal Failure |
| M | 2 | current_smoker | 0.92 (0.52, 1.63) | 0.768 | 1.16 (0.74, 1.8) | 0.521 | Renal Failure |
| M | 2 | total_chol | 1.23 (0.98, 1.54) | 0.074 | 1.07 (0.88, 1.3) | 0.513 | Renal Failure |
| M | 2 | hdl | 0.56 (0.22, 1.45) | 0.233 | 0.84 (0.54, 1.31) | 0.447 | Renal Failure |
| M | 2 | I(sbp/10) | 1.3 (1.1, 1.54) | 0.002 | 1.22 (1.05, 1.4) | 0.007 | Renal Failure |
| M | 2 | ethnicasian | 3.37 (1.18, 9.6) | 0.023 | 1.65 (0.58, 4.64) | 0.342 | Renal Failure |
| M | 2 | ethnicother | 1.13 (0.63, 2.01) | 0.681 | 1.73 (0.84, 3.56) | 0.139 | Renal Failure |
| M | 2 | I(hba1c/10) | 1.11 (0.96, 1.28) | 0.177 | 1.08 (0.95, 1.22) | 0.23 | Renal Failure |
| M | 2 | cv_disease | 2.5 (1.41, 4.43) | 0.002 | 2.08 (1.28, 3.37) | 0.003 | Renal Failure |
| M | 2 | bmi | 0.97 (0.93, 1.02) | 0.273 | 0.98 (0.95, 1.02) | 0.318 | Renal Failure |
| M | 3 | (Intercept) | -11.57 (-16.69, -6.45) | NA | -12.03 (-16.09, -7.96) | NA | Renal Failure |
| M | 3 | agenow10 | 1.88 (0.88, 4.02) | 0.101 | 1.88 (1.04, 3.41) | 0.038 | Renal Failure |
| M | 3 | agedx10 | 0.56 (0.25, 1.22) | 0.145 | 0.56 (0.3, 1.03) | 0.062 | Renal Failure |
| M | 3 | simd_b | 0.81 (0.42, 1.53) | 0.509 | 0.84 (0.5, 1.41) | 0.505 | Renal Failure |
| M | 3 | current_smoker | 1.43 (0.68, 3.01) | 0.34 | 1.38 (0.72, 2.65) | 0.324 | Renal Failure |
| M | 3 | total_chol | 0.69 (0.47, 1.01) | 0.058 | 0.84 (0.63, 1.11) | 0.219 | Renal Failure |
| M | 3 | hdl | 0.79 (0.23, 2.77) | 0.716 | 0.87 (0.47, 1.6) | 0.656 | Renal Failure |
| M | 3 | I(sbp/10) | 1.36 (1.08, 1.7) | 0.008 | 1.37 (1.15, 1.64) | <0.001 | Renal Failure |
| M | 3 | ethnicasian | 0 | 0.978 | 0.6 (0.08, 4.54) | 0.619 | Renal Failure |
| M | 3 | ethnicother | 1.32 (0.65, 2.68) | 0.438 | 1.24 (0.36, 4.29) | 0.73 | Renal Failure |
| M | 3 | I(hba1c/10) | 1.14 (0.94, 1.4) | 0.19 | 1.12 (0.95, 1.32) | 0.167 | Renal Failure |
| M | 3 | cv_disease | 0.78 (0.27, 2.25) | 0.643 | 0.75 (0.29, 1.93) | 0.556 | Renal Failure |
| M | 3 | bmi | 0.98 (0.92, 1.04) | 0.513 | 0.97 (0.92, 1.02) | 0.194 | Renal Failure |
| M | 4 | (Intercept) | -12.2 (-16.75, -7.65) | NA | -10.39 (-14.17, -6.61) | NA | Renal Failure |
| M | 4 | agenow10 | 1.68 (0.86, 3.28) | 0.131 | 1.56 (0.89, 2.75) | 0.119 | Renal Failure |
| M | 4 | agedx10 | 0.71 (0.35, 1.43) | 0.338 | 0.64 (0.36, 1.16) | 0.141 | Renal Failure |
| M | 4 | simd_b | 0.69 (0.39, 1.23) | 0.207 | 0.68 (0.42, 1.11) | 0.124 | Renal Failure |
| M | 4 | current_smoker | 1.26 (0.62, 2.56) | 0.528 | 1.2 (0.64, 2.24) | 0.57 | Renal Failure |
| M | 4 | total_chol | 1 (0.73, 1.36) | 0.993 | 0.93 (0.72, 1.21) | 0.591 | Renal Failure |
| M | 4 | hdl | 0.36 (0.11, 1.19) | 0.094 | 0.86 (0.48, 1.54) | 0.606 | Renal Failure |
| M | 4 | I(sbp/10) | 1.37 (1.12, 1.67) | 0.002 | 1.21 (1.02, 1.44) | 0.033 | Renal Failure |
| M | 4 | ethnicasian | 0.99 (0.13, 7.48) | 0.996 | 0.77 (0.13, 4.71) | 0.778 | Renal Failure |
| M | 4 | ethnicother | 0.38 (0.15, 0.96) | 0.04 | 0.59 (0.11, 3.04) | 0.522 | Renal Failure |
| M | 4 | I(hba1c/10) | 1.07 (0.89, 1.29) | 0.445 | 0.99 (0.84, 1.18) | 0.932 | Renal Failure |
| M | 4 | cv_disease | 1.56 (0.71, 3.45) | 0.268 | 1.51 (0.75, 3.04) | 0.252 | Renal Failure |
| M | 4 | bmi | 0.98 (0.92, 1.04) | 0.427 | 1 (0.95, 1.04) | 0.895 | Renal Failure |
| M | 5 | (Intercept) | -18.66 (-23.95, -13.37) | NA | -16.49 (-20.76, -12.23) | NA | Renal Failure |
| M | 5 | agenow10 | 3.82 (1.83, 7.99) | <0.001 | 2.98 (1.64, 5.41) | <0.001 | Renal Failure |
| M | 5 | agedx10 | 0.39 (0.18, 0.84) | 0.016 | 0.47 (0.25, 0.87) | 0.016 | Renal Failure |
| M | 5 | simd_b | 0.73 (0.37, 1.44) | 0.357 | 0.86 (0.5, 1.48) | 0.58 | Renal Failure |
| M | 5 | current_smoker | 1.78 (0.77, 4.13) | 0.179 | 1.36 (0.62, 3) | 0.446 | Renal Failure |
| M | 5 | total_chol | 1.26 (0.9, 1.76) | 0.17 | 0.97 (0.72, 1.29) | 0.818 | Renal Failure |
| M | 5 | hdl | 0.78 (0.22, 2.74) | 0.697 | 1.01 (0.55, 1.86) | 0.973 | Renal Failure |
| M | 5 | I(sbp/10) | 1.37 (1.08, 1.73) | 0.009 | 1.35 (1.11, 1.63) | 0.002 | Renal Failure |
| M | 5 | ethnicasian | 0.98 (0.13, 7.36) | 0.987 | 1.03 (0.25, 4.29) | 0.964 | Renal Failure |
| M | 5 | ethnicother | 0.39 (0.14, 1.1) | 0.074 | 0.56 (0.09, 3.55) | 0.533 | Renal Failure |
| M | 5 | I(hba1c/10) | 1.02 (0.81, 1.28) | 0.87 | 1.08 (0.9, 1.3) | 0.425 | Renal Failure |
| M | 5 | cv_disease | 1.77 (0.66, 4.71) | 0.256 | 1.23 (0.51, 2.96) | 0.642 | Renal Failure |
| M | 5 | bmi | 1.06 (1, 1.12) | 0.043 | 1.04 (0.98, 1.09) | 0.174 | Renal Failure |
| F | 1 | (Intercept) | -12.57 (-16.88, -8.26) | NA | -12.47 (-15.87, -9.06) | NA | Renal Failure |
| F | 1 | agenow10 | 1.83 (0.94, 3.56) | 0.075 | 1.67 (0.96, 2.89) | 0.067 | Renal Failure |
| F | 1 | agedx10 | 0.64 (0.32, 1.29) | 0.213 | 0.62 (0.35, 1.11) | 0.107 | Renal Failure |
| F | 1 | simd_b | 1.36 (0.77, 2.43) | 0.291 | 1.27 (0.79, 2.04) | 0.332 | Renal Failure |
| F | 1 | current_smoker | 1.36 (0.72, 2.56) | 0.338 | 1.62 (0.94, 2.8) | 0.082 | Renal Failure |
| F | 1 | total_chol | 1.07 (0.81, 1.42) | 0.635 | 1.09 (0.87, 1.36) | 0.468 | Renal Failure |
| F | 1 | hdl | 0.34 (0.11, 1.03) | 0.057 | 0.65 (0.38, 1.12) | 0.122 | Renal Failure |
| F | 1 | I(sbp/10) | 1.27 (1.04, 1.56) | 0.02 | 1.25 (1.07, 1.47) | 0.006 | Renal Failure |
| F | 1 | ethnicasian | 6.21 (1.8, 21.44) | 0.004 | 2.28 (0.72, 7.22) | 0.16 | Renal Failure |
| F | 1 | ethnicother | 0.63 (0.25, 1.6) | 0.33 | 0.5 (0.06, 3.93) | 0.506 | Renal Failure |
| F | 1 | I(hba1c/10) | 0.98 (0.81, 1.19) | 0.839 | 1.08 (0.93, 1.24) | 0.31 | Renal Failure |
| F | 1 | cv_disease | 1.36 (0.56, 3.26) | 0.496 | 1.29 (0.61, 2.75) | 0.505 | Renal Failure |
| F | 1 | bmi | 1.01 (0.97, 1.06) | 0.564 | 1 (0.96, 1.04) | 0.943 | Renal Failure |
| F | 2 | (Intercept) | -9.32 (-13.99, -4.65) | NA | -9.09 (-12.87, -5.31) | NA | Renal Failure |
| F | 2 | agenow10 | 2.7 (1.36, 5.36) | 0.004 | 2.14 (1.25, 3.68) | 0.006 | Renal Failure |
| F | 2 | agedx10 | 0.37 (0.18, 0.76) | 0.007 | 0.44 (0.25, 0.77) | 0.004 | Renal Failure |
| F | 2 | simd_b | 1.36 (0.74, 2.49) | 0.326 | 1.24 (0.77, 2.01) | 0.38 | Renal Failure |
| F | 2 | current_smoker | 1 (0.48, 2.05) | 0.992 | 0.83 (0.44, 1.56) | 0.567 | Renal Failure |
| F | 2 | total_chol | 1.22 (0.91, 1.64) | 0.186 | 1.07 (0.85, 1.35) | 0.543 | Renal Failure |
| F | 2 | hdl | 0.38 (0.12, 1.17) | 0.093 | 0.85 (0.49, 1.48) | 0.563 | Renal Failure |
| F | 2 | I(sbp/10) | 1.12 (0.89, 1.4) | 0.331 | 1.03 (0.86, 1.24) | 0.727 | Renal Failure |
| F | 2 | ethnicasian | 1.3 (0.17, 9.91) | 0.803 | 0.83 (0.11, 6.28) | 0.853 | Renal Failure |
| F | 2 | ethnicother | 0.68 (0.29, 1.62) | 0.386 | 0.96 (0.26, 3.6) | 0.952 | Renal Failure |
| F | 2 | I(hba1c/10) | 1.01 (0.82, 1.25) | 0.896 | 1.11 (0.95, 1.29) | 0.179 | Renal Failure |
| F | 2 | cv_disease | 1.17 (0.41, 3.34) | 0.776 | 0.92 (0.36, 2.35) | 0.87 | Renal Failure |
| F | 2 | bmi | 0.97 (0.92, 1.02) | 0.185 | 0.99 (0.95, 1.03) | 0.539 | Renal Failure |
| F | 3 | (Intercept) | -11.78 (-17.33, -6.23) | NA | -10.74 (-15.09, -6.4) | NA | Renal Failure |
| F | 3 | agenow10 | 2.42 (1.05, 5.62) | 0.039 | 2 (1.02, 3.91) | 0.044 | Renal Failure |
| F | 3 | agedx10 | 0.34 (0.14, 0.83) | 0.018 | 0.41 (0.2, 0.84) | 0.015 | Renal Failure |
| F | 3 | simd_b | 0.85 (0.4, 1.8) | 0.675 | 0.96 (0.53, 1.75) | 0.891 | Renal Failure |
| F | 3 | current_smoker | 2.29 (1.03, 5.08) | 0.042 | 2.01 (1.03, 3.92) | 0.04 | Renal Failure |
| F | 3 | total_chol | 1.14 (0.79, 1.65) | 0.493 | 1.04 (0.77, 1.4) | 0.801 | Renal Failure |
| F | 3 | hdl | 0.36 (0.09, 1.42) | 0.146 | 0.88 (0.45, 1.7) | 0.706 | Renal Failure |
| F | 3 | I(sbp/10) | 1.42 (1.1, 1.84) | 0.007 | 1.24 (1.02, 1.52) | 0.032 | Renal Failure |
| F | 3 | ethnicasian | 0 | 0.981 | 0.92 (0.14, 6.09) | 0.93 | Renal Failure |
| F | 3 | ethnicother | 0.97 (0.39, 2.41) | 0.949 | 0.01 | 0.983 | Renal Failure |
| F | 3 | I(hba1c/10) | 1.02 (0.79, 1.31) | 0.893 | 1.06 (0.87, 1.3) | 0.546 | Renal Failure |
| F | 3 | cv_disease | 1.7 (0.5, 5.8) | 0.398 | 1.82 (0.7, 4.77) | 0.222 | Renal Failure |
| F | 3 | bmi | 0.98 (0.92, 1.04) | 0.468 | 0.98 (0.93, 1.03) | 0.516 | Renal Failure |
| F | 4 | (Intercept) | -15.51 (-21.81, -9.2) | NA | -13.54 (-18.34, -8.73) | NA | Renal Failure |
| F | 4 | agenow10 | 2.31 (0.88, 6.06) | 0.089 | 2.1 (0.98, 4.51) | 0.055 | Renal Failure |
| F | 4 | agedx10 | 0.35 (0.12, 0.97) | 0.043 | 0.37 (0.17, 0.82) | 0.014 | Renal Failure |
| F | 4 | simd_b | 0.55 (0.22, 1.36) | 0.193 | 0.65 (0.32, 1.32) | 0.23 | Renal Failure |
| F | 4 | current_smoker | 1.56 (0.54, 4.49) | 0.406 | 1.69 (0.74, 3.84) | 0.212 | Renal Failure |
| F | 4 | total_chol | 1.13 (0.73, 1.74) | 0.592 | 0.87 (0.62, 1.23) | 0.438 | Renal Failure |
| F | 4 | hdl | 0.97 (0.23, 4.08) | 0.97 | 1.13 (0.55, 2.32) | 0.743 | Renal Failure |
| F | 4 | I(sbp/10) | 1.54 (1.15, 2.04) | 0.003 | 1.44 (1.15, 1.81) | 0.002 | Renal Failure |
| F | 4 | ethnicasian | 4.56 (0.97, 21.42) | 0.055 | 2.29 (0.68, 7.8) | 0.183 | Renal Failure |
| F | 4 | ethnicother | 0.41 (0.09, 1.79) | 0.236 | 0 | 0.987 | Renal Failure |
| F | 4 | I(hba1c/10) | 1.18 (0.91, 1.54) | 0.218 | 1.24 (1.03, 1.5) | 0.025 | Renal Failure |
| F | 4 | cv_disease | 2.11 (0.47, 9.55) | 0.331 | 1.14 (0.26, 4.93) | 0.858 | Renal Failure |
| F | 4 | bmi | 1.01 (0.94, 1.07) | 0.829 | 1 (0.95, 1.06) | 0.866 | Renal Failure |
| F | 5 | (Intercept) | -14.7 (-22.84, -6.57) | NA | -9.53 (-15.24, -3.82) | NA | Renal Failure |
| F | 5 | agenow10 | 2.49 (0.77, 8.11) | 0.129 | 1.66 (0.72, 3.84) | 0.236 | Renal Failure |
| F | 5 | agedx10 | 0.39 (0.11, 1.34) | 0.133 | 0.49 (0.21, 1.19) | 0.116 | Renal Failure |
| F | 5 | simd_b | 1.29 (0.45, 3.69) | 0.634 | 1.26 (0.6, 2.65) | 0.542 | Renal Failure |
| F | 5 | current_smoker | 0.68 (0.09, 5.34) | 0.716 | 1.61 (0.55, 4.73) | 0.382 | Renal Failure |
| F | 5 | total_chol | 0.9 (0.51, 1.61) | 0.724 | 1.18 (0.82, 1.7) | 0.371 | Renal Failure |
| F | 5 | hdl | 0.56 (0.09, 3.33) | 0.52 | 0.94 (0.42, 2.07) | 0.875 | Renal Failure |
| F | 5 | I(sbp/10) | 1.61 (1.11, 2.34) | 0.011 | 1.18 (0.9, 1.54) | 0.238 | Renal Failure |
| F | 5 | ethnicasian | 4.49 (0.88, 22.83) | 0.07 | 2.1 (0.61, 7.26) | 0.238 | Renal Failure |
| F | 5 | ethnicother | 0.98 (0.27, 3.65) | 0.981 | 1 (0.14, 7.28) | 0.997 | Renal Failure |
| F | 5 | I(hba1c/10) | 1 (0.69, 1.46) | 0.993 | 1.03 (0.8, 1.33) | 0.82 | Renal Failure |
| F | 5 | cv_disease | 1.11 (0.14, 8.86) | 0.925 | 0.68 (0.09, 5.16) | 0.713 | Renal Failure |
| F | 5 | bmi | 0.99 (0.91, 1.09) | 0.887 | 0.95 (0.89, 1.02) | 0.185 | Renal Failure |
| M | 1 | (Intercept) | -9.3 (-10.34, -8.26) | NA | -9.07 (-9.93, -8.22) | NA | Stroke |
| M | 1 | agenow10 | 1.64 (1.43, 1.87) | <0.001 | 1.64 (1.47, 1.82) | <0.001 | Stroke |
| M | 1 | agedx10 | 0.97 (0.84, 1.11) | 0.635 | 0.93 (0.83, 1.04) | 0.196 | Stroke |
| M | 1 | simd_b | 0.93 (0.83, 1.03) | 0.163 | 0.92 (0.84, 1) | 0.053 | Stroke |
| M | 1 | current_smoker | 1.48 (1.31, 1.67) | <0.001 | 1.48 (1.34, 1.64) | <0.001 | Stroke |
| M | 1 | total_chol | 1.03 (0.97, 1.09) | 0.316 | 1.02 (0.97, 1.07) | 0.442 | Stroke |
| M | 1 | hdl | 0.97 (0.8, 1.18) | 0.772 | 1 (0.9, 1.1) | 0.925 | Stroke |
| M | 1 | I(sbp/10) | 1.11 (1.07, 1.16) | <0.001 | 1.13 (1.09, 1.17) | <0.001 | Stroke |
| M | 1 | ethnicasian | 0.75 (0.46, 1.21) | 0.239 | 0.78 (0.51, 1.19) | 0.248 | Stroke |
| M | 1 | ethnicother | 1.05 (0.91, 1.21) | 0.536 | 1.08 (0.82, 1.43) | 0.569 | Stroke |
| M | 1 | I(egfr/10) | 0.95 (0.91, 0.99) | 0.009 | 0.94 (0.91, 0.97) | <0.001 | Stroke |
| M | 1 | I(hba1c/10) | 1.03 (1, 1.07) | 0.074 | 1.04 (1.01, 1.07) | 0.005 | Stroke |
| M | 1 | cv_disease | 1.43 (1.24, 1.64) | <0.001 | 1.53 (1.36, 1.71) | <0.001 | Stroke |
| M | 1 | bmi | 0.99 (0.98, 1) | 0.067 | 0.99 (0.98, 1) | 0.004 | Stroke |
| M | 2 | (Intercept) | -10.92 (-12.02, -9.82) | NA | -10.38 (-11.25, -9.51) | NA | Stroke |
| M | 2 | agenow10 | 1.68 (1.46, 1.93) | <0.001 | 1.48 (1.33, 1.65) | <0.001 | Stroke |
| M | 2 | agedx10 | 1.04 (0.9, 1.21) | 0.575 | 1.14 (1.02, 1.28) | 0.022 | Stroke |
| M | 2 | simd_b | 0.98 (0.87, 1.09) | 0.699 | 0.96 (0.88, 1.06) | 0.435 | Stroke |
| M | 2 | current_smoker | 1.55 (1.35, 1.77) | <0.001 | 1.44 (1.28, 1.62) | <0.001 | Stroke |
| M | 2 | total_chol | 1.09 (1.02, 1.16) | 0.008 | 1.07 (1.02, 1.12) | 0.009 | Stroke |
| M | 2 | hdl | 1.18 (0.97, 1.44) | 0.104 | 1.07 (0.97, 1.18) | 0.185 | Stroke |
| M | 2 | I(sbp/10) | 1.12 (1.07, 1.17) | <0.001 | 1.12 (1.08, 1.16) | <0.001 | Stroke |
| M | 2 | ethnicasian | 1.11 (0.69, 1.81) | 0.665 | 1.09 (0.76, 1.57) | 0.626 | Stroke |
| M | 2 | ethnicother | 1.14 (1, 1.3) | 0.055 | 1.27 (0.99, 1.64) | 0.064 | Stroke |
| M | 2 | I(egfr/10) | 0.92 (0.88, 0.96) | <0.001 | 0.93 (0.9, 0.96) | <0.001 | Stroke |
| M | 2 | I(hba1c/10) | 1.07 (1.03, 1.11) | 0.001 | 1.07 (1.03, 1.1) | <0.001 | Stroke |
| M | 2 | cv_disease | 1.69 (1.47, 1.95) | <0.001 | 1.61 (1.43, 1.8) | <0.001 | Stroke |
| M | 2 | bmi | 1 (0.99, 1.01) | 0.753 | 1 (0.99, 1.01) | 0.548 | Stroke |
| M | 3 | (Intercept) | -10.26 (-11.44, -9.07) | NA | -10.31 (-11.27, -9.35) | NA | Stroke |
| M | 3 | agenow10 | 1.75 (1.5, 2.03) | <0.001 | 1.7 (1.51, 1.91) | <0.001 | Stroke |
| M | 3 | agedx10 | 1.01 (0.86, 1.17) | 0.944 | 1.01 (0.9, 1.14) | 0.838 | Stroke |
| M | 3 | simd_b | 1.05 (0.93, 1.19) | 0.423 | 0.99 (0.9, 1.09) | 0.805 | Stroke |
| M | 3 | current_smoker | 1.56 (1.35, 1.81) | <0.001 | 1.44 (1.27, 1.64) | <0.001 | Stroke |
| M | 3 | total_chol | 1.02 (0.95, 1.09) | 0.586 | 0.99 (0.94, 1.05) | 0.809 | Stroke |
| M | 3 | hdl | 0.94 (0.75, 1.17) | 0.573 | 1.17 (1.06, 1.3) | 0.002 | Stroke |
| M | 3 | I(sbp/10) | 1.08 (1.03, 1.13) | 0.001 | 1.09 (1.05, 1.13) | <0.001 | Stroke |
| M | 3 | ethnicasian | 0.99 (0.58, 1.68) | 0.96 | 1.04 (0.72, 1.5) | 0.829 | Stroke |
| M | 3 | ethnicother | 0.99 (0.86, 1.14) | 0.862 | 1.03 (0.8, 1.34) | 0.801 | Stroke |
| M | 3 | I(egfr/10) | 0.92 (0.88, 0.96) | <0.001 | 0.93 (0.9, 0.96) | <0.001 | Stroke |
| M | 3 | I(hba1c/10) | 1.07 (1.03, 1.12) | <0.001 | 1.07 (1.03, 1.1) | <0.001 | Stroke |
| M | 3 | cv_disease | 1.51 (1.29, 1.76) | <0.001 | 1.56 (1.37, 1.77) | <0.001 | Stroke |
| M | 3 | bmi | 1.01 (0.99, 1.02) | 0.406 | 1 (0.99, 1.01) | 0.394 | Stroke |
| M | 4 | (Intercept) | -10.55 (-11.79, -9.32) | NA | -10.95 (-11.95, -9.95) | NA | Stroke |
| M | 4 | agenow10 | 1.65 (1.41, 1.93) | <0.001 | 1.63 (1.44, 1.85) | <0.001 | Stroke |
| M | 4 | agedx10 | 1.13 (0.96, 1.32) | 0.141 | 1.12 (0.98, 1.27) | 0.092 | Stroke |
| M | 4 | simd_b | 0.87 (0.77, 0.99) | 0.032 | 0.93 (0.84, 1.03) | 0.177 | Stroke |
| M | 4 | current_smoker | 1.54 (1.3, 1.81) | <0.001 | 1.51 (1.29, 1.76) | <0.001 | Stroke |
| M | 4 | total_chol | 0.97 (0.9, 1.05) | 0.438 | 1 (0.95, 1.06) | 0.899 | Stroke |
| M | 4 | hdl | 0.97 (0.77, 1.22) | 0.789 | 1.07 (0.95, 1.2) | 0.254 | Stroke |
| M | 4 | I(sbp/10) | 1.1 (1.05, 1.15) | <0.001 | 1.12 (1.08, 1.17) | <0.001 | Stroke |
| M | 4 | ethnicasian | 0.32 (0.12, 0.86) | 0.025 | 0.95 (0.62, 1.46) | 0.809 | Stroke |
| M | 4 | ethnicother | 1.09 (0.95, 1.26) | 0.222 | 1 (0.73, 1.35) | 0.976 | Stroke |
| M | 4 | I(egfr/10) | 0.95 (0.91, 1) | 0.038 | 0.94 (0.9, 0.98) | 0.001 | Stroke |
| M | 4 | I(hba1c/10) | 1.06 (1.01, 1.11) | 0.01 | 1.06 (1.02, 1.1) | 0.002 | Stroke |
| M | 4 | cv_disease | 1.45 (1.23, 1.71) | <0.001 | 1.41 (1.23, 1.62) | <0.001 | Stroke |
| M | 4 | bmi | 1 (0.99, 1.01) | 0.857 | 1 (0.99, 1.01) | 0.714 | Stroke |
| M | 5 | (Intercept) | -10.93 (-12.35, -9.51) | NA | -10.87 (-12.05, -9.7) | NA | Stroke |
| M | 5 | agenow10 | 1.95 (1.64, 2.32) | <0.001 | 1.94 (1.68, 2.23) | <0.001 | Stroke |
| M | 5 | agedx10 | 0.97 (0.81, 1.15) | 0.718 | 0.96 (0.83, 1.11) | 0.573 | Stroke |
| M | 5 | simd_b | 1.03 (0.9, 1.19) | 0.663 | 1.04 (0.93, 1.17) | 0.492 | Stroke |
| M | 5 | current_smoker | 1.46 (1.19, 1.8) | <0.001 | 1.42 (1.19, 1.69) | <0.001 | Stroke |
| M | 5 | total_chol | 0.99 (0.91, 1.08) | 0.856 | 0.98 (0.92, 1.05) | 0.556 | Stroke |
| M | 5 | hdl | 0.93 (0.72, 1.21) | 0.608 | 0.97 (0.85, 1.11) | 0.682 | Stroke |
| M | 5 | I(sbp/10) | 1.12 (1.06, 1.18) | <0.001 | 1.13 (1.08, 1.18) | <0.001 | Stroke |
| M | 5 | ethnicasian | 0.99 (0.63, 1.56) | 0.963 | 0.95 (0.66, 1.37) | 0.779 | Stroke |
| M | 5 | ethnicother | 1.16 (0.99, 1.36) | 0.065 | 1.15 (0.84, 1.59) | 0.379 | Stroke |
| M | 5 | I(egfr/10) | 0.97 (0.92, 1.02) | 0.255 | 0.95 (0.91, 0.99) | 0.028 | Stroke |
| M | 5 | I(hba1c/10) | 1.04 (0.98, 1.09) | 0.191 | 1.04 (0.99, 1.09) | 0.1 | Stroke |
| M | 5 | cv_disease | 1.57 (1.29, 1.9) | <0.001 | 1.6 (1.37, 1.88) | <0.001 | Stroke |
| M | 5 | bmi | 0.99 (0.97, 1.01) | 0.21 | 0.99 (0.98, 1.01) | 0.426 | Stroke |
| F | 1 | (Intercept) | -9.66 (-10.72, -8.61) | NA | -9.37 (-10.22, -8.52) | NA | Stroke |
| F | 1 | agenow10 | 1.77 (1.54, 2.03) | <0.001 | 1.6 (1.44, 1.79) | <0.001 | Stroke |
| F | 1 | agedx10 | 0.85 (0.74, 0.98) | 0.026 | 0.94 (0.84, 1.05) | 0.28 | Stroke |
| F | 1 | simd_b | 0.95 (0.85, 1.07) | 0.412 | 0.97 (0.89, 1.07) | 0.554 | Stroke |
| F | 1 | current_smoker | 1.24 (1.08, 1.41) | 0.002 | 1.3 (1.17, 1.46) | <0.001 | Stroke |
| F | 1 | total_chol | 1.05 (0.99, 1.12) | 0.076 | 1.01 (0.97, 1.06) | 0.532 | Stroke |
| F | 1 | hdl | 0.93 (0.77, 1.11) | 0.409 | 0.99 (0.9, 1.09) | 0.867 | Stroke |
| F | 1 | I(sbp/10) | 1.13 (1.09, 1.18) | <0.001 | 1.11 (1.07, 1.15) | <0.001 | Stroke |
| F | 1 | ethnicasian | 0.6 (0.27, 1.35) | 0.218 | 0.79 (0.49, 1.27) | 0.317 | Stroke |
| F | 1 | ethnicother | 1.03 (0.89, 1.2) | 0.668 | 0.96 (0.66, 1.4) | 0.829 | Stroke |
| F | 1 | I(egfr/10) | 0.91 (0.88, 0.95) | <0.001 | 0.92 (0.89, 0.95) | <0.001 | Stroke |
| F | 1 | I(hba1c/10) | 1.11 (1.07, 1.15) | <0.001 | 1.12 (1.08, 1.15) | <0.001 | Stroke |
| F | 1 | cv_disease | 1.83 (1.57, 2.12) | <0.001 | 1.77 (1.56, 1.99) | <0.001 | Stroke |
| F | 1 | bmi | 0.99 (0.99, 1) | 0.272 | 1 (0.99, 1.01) | 0.537 | Stroke |
| F | 2 | (Intercept) | -10.42 (-11.57, -9.27) | NA | -10.04 (-10.97, -9.11) | NA | Stroke |
| F | 2 | agenow10 | 2.1 (1.82, 2.42) | <0.001 | 1.85 (1.64, 2.08) | <0.001 | Stroke |
| F | 2 | agedx10 | 0.86 (0.74, 1) | 0.05 | 0.93 (0.82, 1.05) | 0.227 | Stroke |
| F | 2 | simd_b | 0.97 (0.86, 1.09) | 0.584 | 0.93 (0.84, 1.02) | 0.132 | Stroke |
| F | 2 | current_smoker | 1.58 (1.37, 1.82) | <0.001 | 1.48 (1.31, 1.66) | <0.001 | Stroke |
| F | 2 | total_chol | 0.97 (0.91, 1.03) | 0.332 | 0.94 (0.9, 0.99) | 0.027 | Stroke |
| F | 2 | hdl | 0.89 (0.73, 1.07) | 0.208 | 0.94 (0.85, 1.04) | 0.223 | Stroke |
| F | 2 | I(sbp/10) | 1.09 (1.04, 1.14) | <0.001 | 1.11 (1.08, 1.15) | <0.001 | Stroke |
| F | 2 | ethnicasian | 1.07 (0.57, 2.01) | 0.835 | 1.11 (0.73, 1.68) | 0.631 | Stroke |
| F | 2 | ethnicother | 1.12 (0.98, 1.29) | 0.108 | 1.14 (0.89, 1.45) | 0.3 | Stroke |
| F | 2 | I(egfr/10) | 0.96 (0.92, 1) | 0.031 | 0.96 (0.92, 0.99) | 0.012 | Stroke |
| F | 2 | I(hba1c/10) | 1.08 (1.04, 1.13) | <0.001 | 1.09 (1.05, 1.12) | <0.001 | Stroke |
| F | 2 | cv_disease | 1.65 (1.4, 1.94) | <0.001 | 1.67 (1.46, 1.9) | <0.001 | Stroke |
| F | 2 | bmi | 1 (0.99, 1.01) | 0.638 | 0.99 (0.98, 1) | 0.057 | Stroke |
| F | 3 | (Intercept) | -11.25 (-12.55, -9.95) | NA | -10.46 (-11.5, -9.41) | NA | Stroke |
| F | 3 | agenow10 | 2.04 (1.73, 2.41) | <0.001 | 1.83 (1.6, 2.09) | <0.001 | Stroke |
| F | 3 | agedx10 | 0.96 (0.81, 1.14) | 0.676 | 1 (0.88, 1.15) | 0.98 | Stroke |
| F | 3 | simd_b | 1.04 (0.91, 1.2) | 0.529 | 0.98 (0.88, 1.09) | 0.691 | Stroke |
| F | 3 | current_smoker | 1.59 (1.34, 1.9) | <0.001 | 1.62 (1.4, 1.87) | <0.001 | Stroke |
| F | 3 | total_chol | 0.99 (0.92, 1.06) | 0.752 | 1.01 (0.95, 1.07) | 0.836 | Stroke |
| F | 3 | hdl | 0.77 (0.62, 0.95) | 0.015 | 0.96 (0.85, 1.08) | 0.497 | Stroke |
| F | 3 | I(sbp/10) | 1.13 (1.08, 1.19) | <0.001 | 1.1 (1.05, 1.14) | <0.001 | Stroke |
| F | 3 | ethnicasian | 1.08 (0.53, 2.18) | 0.84 | 1.25 (0.85, 1.84) | 0.25 | Stroke |
| F | 3 | ethnicother | 1.05 (0.9, 1.23) | 0.502 | 0.94 (0.71, 1.24) | 0.649 | Stroke |
| F | 3 | I(egfr/10) | 0.97 (0.92, 1.01) | 0.164 | 0.93 (0.89, 0.96) | <0.001 | Stroke |
| F | 3 | I(hba1c/10) | 1.07 (1.01, 1.12) | 0.012 | 1.1 (1.06, 1.14) | <0.001 | Stroke |
| F | 3 | cv_disease | 1.51 (1.25, 1.84) | <0.001 | 1.62 (1.39, 1.89) | <0.001 | Stroke |
| F | 3 | bmi | 0.99 (0.98, 1) | 0.03 | 0.99 (0.98, 1) | 0.017 | Stroke |
| F | 4 | (Intercept) | -11.15 (-12.52, -9.78) | NA | -10.71 (-11.83, -9.59) | NA | Stroke |
| F | 4 | agenow10 | 1.97 (1.65, 2.34) | <0.001 | 1.87 (1.63, 2.16) | <0.001 | Stroke |
| F | 4 | agedx10 | 1.04 (0.87, 1.23) | 0.699 | 1.03 (0.9, 1.19) | 0.667 | Stroke |
| F | 4 | simd_b | 0.97 (0.85, 1.12) | 0.721 | 0.94 (0.84, 1.06) | 0.315 | Stroke |
| F | 4 | current_smoker | 1.92 (1.59, 2.31) | <0.001 | 1.73 (1.47, 2.03) | <0.001 | Stroke |
| F | 4 | total_chol | 1.01 (0.94, 1.09) | 0.74 | 0.98 (0.93, 1.04) | 0.6 | Stroke |
| F | 4 | hdl | 1.03 (0.83, 1.28) | 0.769 | 0.95 (0.85, 1.07) | 0.442 | Stroke |
| F | 4 | I(sbp/10) | 1.05 (1, 1.11) | 0.065 | 1.08 (1.03, 1.13) | <0.001 | Stroke |
| F | 4 | ethnicasian | 1 (0.51, 1.95) | 0.999 | 1.03 (0.66, 1.6) | 0.91 | Stroke |
| F | 4 | ethnicother | 1.11 (0.94, 1.3) | 0.213 | 1.05 (0.79, 1.39) | 0.756 | Stroke |
| F | 4 | I(egfr/10) | 0.89 (0.85, 0.94) | <0.001 | 0.9 (0.87, 0.94) | <0.001 | Stroke |
| F | 4 | I(hba1c/10) | 1.17 (1.12, 1.23) | <0.001 | 1.13 (1.08, 1.17) | <0.001 | Stroke |
| F | 4 | cv_disease | 1.5 (1.22, 1.84) | <0.001 | 1.66 (1.41, 1.95) | <0.001 | Stroke |
| F | 4 | bmi | 1 (0.98, 1.01) | 0.511 | 1 (0.99, 1.01) | 0.516 | Stroke |
| F | 5 | (Intercept) | -13.99 (-15.63, -12.35) | NA | -11.89 (-13.25, -10.53) | NA | Stroke |
| F | 5 | agenow10 | 1.84 (1.5, 2.25) | <0.001 | 1.79 (1.52, 2.11) | <0.001 | Stroke |
| F | 5 | agedx10 | 1.25 (1.01, 1.54) | 0.038 | 1.1 (0.93, 1.3) | 0.266 | Stroke |
| F | 5 | simd_b | 0.92 (0.78, 1.09) | 0.335 | 0.95 (0.83, 1.09) | 0.468 | Stroke |
| F | 5 | current_smoker | 1.76 (1.38, 2.25) | <0.001 | 1.48 (1.18, 1.86) | <0.001 | Stroke |
| F | 5 | total_chol | 1.1 (1.01, 1.2) | 0.029 | 1.07 (1, 1.14) | 0.062 | Stroke |
| F | 5 | hdl | 0.86 (0.67, 1.1) | 0.24 | 1.09 (0.95, 1.25) | 0.237 | Stroke |
| F | 5 | I(sbp/10) | 1.16 (1.09, 1.23) | <0.001 | 1.1 (1.05, 1.16) | <0.001 | Stroke |
| F | 5 | ethnicasian | 1.23 (0.7, 2.15) | 0.475 | 1.16 (0.78, 1.73) | 0.454 | Stroke |
| F | 5 | ethnicother | 1.26 (1.05, 1.5) | 0.013 | 1.19 (0.85, 1.65) | 0.306 | Stroke |
| F | 5 | I(egfr/10) | 0.99 (0.93, 1.05) | 0.717 | 0.93 (0.89, 0.97) | 0.003 | Stroke |
| F | 5 | I(hba1c/10) | 1.06 (0.99, 1.12) | 0.081 | 1.08 (1.03, 1.13) | 0.002 | Stroke |
| F | 5 | cv_disease | 1.58 (1.24, 2.02) | <0.001 | 1.87 (1.54, 2.27) | <0.001 | Stroke |
| F | 5 | bmi | 1 (0.99, 1.02) | 0.556 | 1 (0.99, 1.01) | 0.991 | Stroke |
| OR: odds ratio; ORs are from the complete-case analysis; OR imputed (95% CI) and P value (imputed) represent pooled estimates based on Rubin’s rules across 10 imputed datasets; ORs are reported for covariates only, Intercepts are presented on their natural scale; CI: confidence interval; SIMD: Scottish Index of Multiple Deprivation; M: Male; F: Female; agenow10: age at analysis (per 10 year increase); agedx10: age at T2DM diagnosis (per 10 year increase); total_chol: Total cholesterol; hdl: High-density lipoprotein cholesterol; I(sbp/10): Systolic blood pressure (per 10 mmHg increase); ethnicasian: Ethnicity – Asian; ethnicother: Ethnicity – Other; I(egfr/10): Estimated glomerular filtration rate (per 10 mL/min/1.73m² increase); I(hba1c/10): Glycated haemoglobin (HbA1c) (per 10 mmol/mol increase); bmi: Body mass index; cv_disease: History of cardiovascular disease; simd_b: Binary indicator for each SIMD quintile - coded as 0 for the lower decile (e.g., decile 1) and 1 for the higher decile (e.g., decile 2), as the original SIMD variable is in deciles. | | | | | | | |

Table A6. Repeated measure linear / Normal model for costs.

| Sex | SIMD | Parameter | Cost (95% CI) | P value | Cost imputed (95% CI) | P value imputed |
| --- | --- | --- | --- | --- | --- | --- |
| M | 1 | (Intercept) | -7011 (-10086, -3937) | NA | -7533 (-10127, -4940) | NA |
| M | 1 | agenow10 | 2882 (2437, 3326) | <0.001 | 2634 (2262, 3007) | <0.001 |
| M | 1 | agedx10 | -1204 (-1669, -740) | <0.001 | -916 (-1305, -526) | <0.001 |
| M | 1 | simd_b | -123 (-457, 211) | 0.471 | 10 (-276, 295) | 0.948 |
| M | 1 | current_smoker | 968 (593, 1343) | <0.001 | 949 (607, 1291) | <0.001 |
| M | 1 | total_chol | 115 (-62, 291) | 0.203 | 52 (-90, 194) | 0.473 |
| M | 1 | hdl | 904 (329, 1479) | 0.002 | 545 (241, 849) | <0.001 |
| M | 1 | I(sbp/10) | 191 (65, 317) | 0.003 | 236 (131, 341) | <0.001 |
| M | 1 | ethnicasian | -1498 (-2777, -220) | 0.022 | -2 (-1235, 1230) | 0.997 |
| M | 1 | ethnicother | 567 (104, 1029) | 0.016 | 153 (-697, 1003) | 0.722 |
| M | 1 | I(egfr/10) | -155 (-278, -32) | 0.013 | -132 (-237, -26) | 0.015 |
| M | 1 | I(hba1c/10) | 219 (110, 328) | <0.001 | 319 (225, 412) | <0.001 |
| M | 1 | cv_disease | 871 (450, 1292) | <0.001 | 928 (565, 1291) | <0.001 |
| M | 1 | bmi | -10 (-40, 19) | 0.486 | -13 (-38, 13) | 0.33 |
| M | 2 | (Intercept) | -3335 (-6550, -120) | NA | -4178 (-6863, -1494) | NA |
| M | 2 | agenow10 | 2672 (2216, 3129) | <0.001 | 2206 (1828, 2583) | <0.001 |
| M | 2 | agedx10 | -1209 (-1685, -733) | <0.001 | -734 (-1128, -340) | <0.001 |
| M | 2 | simd_b | -372 (-715, -28) | 0.034 | -305 (-594, -16) | 0.038 |
| M | 2 | current_smoker | 1300 (884, 1715) | <0.001 | 1264 (905, 1624) | <0.001 |
| M | 2 | total_chol | 86 (-100, 272) | 0.363 | 35 (-116, 185) | 0.652 |
| M | 2 | hdl | 930 (315, 1545) | 0.003 | 514 (202, 826) | 0.001 |
| M | 2 | I(sbp/10) | 7 (-127, 140) | 0.921 | 98 (-12, 208) | 0.081 |
| M | 2 | ethnicasian | -1697 (-3120, -275) | 0.019 | -651 (-1671, 370) | 0.208 |
| M | 2 | ethnicother | 686 (258, 1114) | 0.002 | -55 (-930, 821) | 0.901 |
| M | 2 | I(egfr/10) | -205 (-331, -80) | 0.001 | -156 (-266, -46) | 0.006 |
| M | 2 | I(hba1c/10) | 189 (70, 307) | 0.002 | 238 (135, 342) | <0.001 |
| M | 2 | cv_disease | 414 (-28, 856) | 0.066 | 573 (200, 946) | 0.003 |
| M | 2 | bmi | 5 (-26, 36) | 0.74 | 9 (-20, 38) | 0.551 |
| M | 3 | (Intercept) | -6549 (-10404, -2694) | NA | -5555 (-8724, -2386) | NA |
| M | 3 | agenow10 | 2082 (1540, 2625) | <0.001 | 1783 (1344, 2222) | <0.001 |
| M | 3 | agedx10 | -467 (-1033, 99) | 0.106 | -192 (-644, 260) | 0.405 |
| M | 3 | simd_b | -385 (-792, 22) | 0.064 | -400 (-735, -65) | 0.019 |
| M | 3 | current_smoker | 1307 (792, 1823) | <0.001 | 1327 (877, 1778) | <0.001 |
| M | 3 | total_chol | -87 (-314, 140) | 0.454 | -87 (-266, 92) | 0.341 |
| M | 3 | hdl | 911 (182, 1641) | 0.014 | 421 (65, 778) | 0.02 |
| M | 3 | I(sbp/10) | 82 (-75, 240) | 0.305 | 132 (4, 260) | 0.043 |
| M | 3 | ethnicasian | 82 (-1555, 1719) | 0.922 | -46 (-1093, 1001) | 0.931 |
| M | 3 | ethnicother | 1050 (561, 1538) | <0.001 | 880 (94, 1666) | 0.028 |
| M | 3 | I(egfr/10) | -296 (-446, -145) | <0.001 | -245 (-374, -116) | <0.001 |
| M | 3 | I(hba1c/10) | 375 (231, 520) | <0.001 | 323 (211, 435) | <0.001 |
| M | 3 | cv_disease | 423 (-112, 959) | 0.121 | 708 (262, 1154) | 0.002 |
| M | 3 | bmi | 68 (29, 106) | <0.001 | 48 (16, 80) | 0.003 |
| M | 4 | (Intercept) | -6785 (-10593, -2977) | NA | -5098 (-8330, -1866) | NA |
| M | 4 | agenow10 | 1767 (1235, 2299) | <0.001 | 1803 (1365, 2240) | <0.001 |
| M | 4 | agedx10 | -160 (-709, 390) | 0.569 | -317 (-772, 138) | 0.172 |
| M | 4 | simd_b | -361 (-763, 41) | 0.079 | -320 (-664, 24) | 0.068 |
| M | 4 | current_smoker | 1316 (768, 1863) | <0.001 | 1293 (823, 1762) | <0.001 |
| M | 4 | total_chol | -26 (-253, 201) | 0.824 | -81 (-271, 109) | 0.401 |
| M | 4 | hdl | -88 (-778, 601) | 0.802 | 443 (57, 830) | 0.025 |
| M | 4 | I(sbp/10) | 134 (-21, 289) | 0.091 | 100 (-29, 228) | 0.128 |
| M | 4 | ethnicasian | -1778 (-3238, -317) | 0.017 | -922 (-2049, 205) | 0.107 |
| M | 4 | ethnicother | 322 (-153, 798) | 0.184 | -193 (-1113, 726) | 0.676 |
| M | 4 | I(egfr/10) | -150 (-300, 0) | 0.05 | -172 (-304, -40) | 0.011 |
| M | 4 | I(hba1c/10) | 413 (271, 555) | <0.001 | 353 (233, 474) | <0.001 |
| M | 4 | cv_disease | -17 (-561, 526) | 0.95 | 246 (-222, 715) | 0.302 |
| M | 4 | bmi | 49 (10, 88) | 0.014 | 43 (10, 77) | 0.01 |
| M | 5 | (Intercept) | -9180 (-13529, -4832) | NA | -10832 (-14496, -7168) | NA |
| M | 5 | agenow10 | 2136 (1532, 2739) | <0.001 | 1952 (1457, 2447) | <0.001 |
| M | 5 | agedx10 | -595 (-1211, 22) | 0.059 | -205 (-712, 301) | 0.427 |
| M | 5 | simd_b | 155 (-305, 615) | 0.508 | -1 (-390, 388) | 0.995 |
| M | 5 | current_smoker | 949 (280, 1618) | 0.005 | 1212 (576, 1848) | <0.001 |
| M | 5 | total_chol | -104 (-363, 155) | 0.431 | -58 (-267, 150) | 0.583 |
| M | 5 | hdl | 1194 (383, 2006) | 0.004 | 115 (-331, 562) | 0.612 |
| M | 5 | I(sbp/10) | 216 (37, 396) | 0.018 | 270 (120, 419) | <0.001 |
| M | 5 | ethnicasian | -1275 (-2561, 11) | 0.052 | -453 (-1682, 777) | 0.459 |
| M | 5 | ethnicother | 378 (-151, 907) | 0.161 | 140 (-801, 1082) | 0.768 |
| M | 5 | I(egfr/10) | -65 (-235, 104) | 0.45 | -45 (-187, 98) | 0.538 |
| M | 5 | I(hba1c/10) | 392 (222, 562) | <0.001 | 448 (308, 589) | <0.001 |
| M | 5 | cv_disease | 763 (127, 1399) | 0.019 | 804 (261, 1348) | 0.004 |
| M | 5 | bmi | 37 (-9, 82) | 0.115 | 48 (7, 89) | 0.023 |
| F | 1 | (Intercept) | -684 (-3680, 2311) | NA | -3338 (-5810, -866) | NA |
| F | 1 | agenow10 | 2392 (1939, 2846) | <0.001 | 2206 (1832, 2581) | <0.001 |
| F | 1 | agedx10 | -1200 (-1673, -728) | <0.001 | -886 (-1272, -501) | <0.001 |
| F | 1 | simd_b | -345 (-688, -2) | 0.048 | -184 (-469, 102) | 0.207 |
| F | 1 | current_smoker | 1123 (739, 1506) | <0.001 | 1146 (818, 1475) | <0.001 |
| F | 1 | total_chol | -163 (-336, 9) | 0.063 | 36 (-106, 179) | 0.618 |
| F | 1 | hdl | 443 (-81, 967) | 0.097 | 404 (97, 711) | 0.01 |
| F | 1 | I(sbp/10) | 122 (-8, 253) | 0.066 | 168 (61, 275) | 0.002 |
| F | 1 | ethnicasian | -1392 (-3103, 318) | 0.111 | -123 (-1694, 1449) | 0.874 |
| F | 1 | ethnicother | 1196 (722, 1671) | <0.001 | 182 (-743, 1107) | 0.697 |
| F | 1 | I(egfr/10) | -451 (-568, -333) | <0.001 | -409 (-507, -310) | <0.001 |
| F | 1 | I(hba1c/10) | 364 (248, 481) | <0.001 | 372 (276, 469) | <0.001 |
| F | 1 | cv_disease | 1045 (573, 1518) | <0.001 | 1461 (1065, 1857) | <0.001 |
| F | 1 | bmi | 18 (-8, 43) | 0.169 | 24 (1, 46) | 0.04 |
| F | 2 | (Intercept) | -8132 (-11505, -4758) | NA | -6793 (-9763, -3823) | NA |
| F | 2 | agenow10 | 2081 (1590, 2573) | <0.001 | 1591 (1181, 2001) | <0.001 |
| F | 2 | agedx10 | -394 (-909, 121) | 0.134 | -37 (-461, 388) | 0.865 |
| F | 2 | simd_b | -223 (-597, 150) | 0.242 | -164 (-482, 155) | 0.314 |
| F | 2 | current_smoker | 1078 (632, 1524) | <0.001 | 912 (512, 1312) | <0.001 |
| F | 2 | total_chol | -81 (-273, 112) | 0.411 | -30 (-195, 136) | 0.725 |
| F | 2 | hdl | 40 (-523, 602) | 0.89 | 126 (-197, 448) | 0.445 |
| F | 2 | I(sbp/10) | 155 (12, 297) | 0.033 | 171 (51, 291) | 0.005 |
| F | 2 | ethnicasian | -793 (-2369, 783) | 0.324 | 223 (-1118, 1564) | 0.738 |
| F | 2 | ethnicother | 1051 (589, 1514) | <0.001 | 538 (-774, 1851) | 0.404 |
| F | 2 | I(egfr/10) | -168 (-297, -38) | 0.011 | -245 (-355, -135) | <0.001 |
| F | 2 | I(hba1c/10) | 391 (259, 522) | <0.001 | 445 (331, 559) | <0.001 |
| F | 2 | cv_disease | 1301 (769, 1832) | <0.001 | 1092 (633, 1552) | <0.001 |
| F | 2 | bmi | 65 (37, 93) | <0.001 | 61 (37, 86) | <0.001 |
| F | 3 | (Intercept) | -758 (-4347, 2831) | NA | -2948 (-6025, 130) | NA |
| F | 3 | agenow10 | 1902 (1366, 2439) | <0.001 | 1631 (1185, 2077) | <0.001 |
| F | 3 | agedx10 | -432 (-987, 123) | 0.127 | -320 (-777, 137) | 0.17 |
| F | 3 | simd_b | 91 (-314, 497) | 0.659 | 1 (-345, 347) | 0.997 |
| F | 3 | current_smoker | 1662 (1137, 2186) | <0.001 | 1535 (1064, 2006) | <0.001 |
| F | 3 | total_chol | 66 (-145, 278) | 0.54 | 245 (67, 423) | 0.007 |
| F | 3 | hdl | -492 (-1112, 128) | 0.12 | 102 (-261, 465) | 0.581 |
| F | 3 | I(sbp/10) | -80 (-236, 76) | 0.314 | 54 (-80, 188) | 0.427 |
| F | 3 | ethnicasian | -43 (-1661, 1576) | 0.959 | -190 (-1332, 953) | 0.742 |
| F | 3 | ethnicother | 708 (230, 1185) | 0.004 | 298 (-575, 1171) | 0.499 |
| F | 3 | I(egfr/10) | -367 (-504, -229) | <0.001 | -376 (-497, -255) | <0.001 |
| F | 3 | I(hba1c/10) | 101 (-49, 251) | 0.185 | 256 (134, 378) | <0.001 |
| F | 3 | cv_disease | 272 (-335, 879) | 0.38 | 967 (456, 1478) | <0.001 |
| F | 3 | bmi | 63 (31, 94) | <0.001 | 47 (20, 74) | <0.001 |
| F | 4 | (Intercept) | -4463 (-8642, -285) | NA | -3761 (-7144, -378) | NA |
| F | 4 | agenow10 | 1666 (1054, 2278) | <0.001 | 1608 (1113, 2103) | <0.001 |
| F | 4 | agedx10 | -120 (-752, 511) | 0.708 | -66 (-572, 440) | 0.798 |
| F | 4 | simd_b | -175 (-635, 284) | 0.455 | -249 (-627, 129) | 0.197 |
| F | 4 | current_smoker | 1603 (973, 2234) | <0.001 | 1465 (938, 1991) | <0.001 |
| F | 4 | total_chol | 34 (-205, 274) | 0.778 | -73 (-263, 118) | 0.454 |
| F | 4 | hdl | 213 (-489, 915) | 0.551 | 499 (75, 924) | 0.021 |
| F | 4 | I(sbp/10) | 47 (-133, 228) | 0.607 | 60 (-86, 206) | 0.421 |
| F | 4 | ethnicasian | -1431 (-3081, 219) | 0.089 | -980 (-2248, 289) | 0.127 |
| F | 4 | ethnicother | 175 (-373, 724) | 0.531 | -400 (-1263, 463) | 0.363 |
| F | 4 | I(egfr/10) | -365 (-523, -207) | <0.001 | -352 (-483, -220) | <0.001 |
| F | 4 | I(hba1c/10) | 214 (46, 383) | 0.013 | 269 (131, 407) | <0.001 |
| F | 4 | cv_disease | 847 (139, 1556) | 0.019 | 1102 (514, 1689) | <0.001 |
| F | 4 | bmi | 80 (44, 117) | <0.001 | 54 (23, 85) | <0.001 |
| F | 5 | (Intercept) | -10032 (-14691, -5372) | NA | -7143 (-11308, -2977) | NA |
| F | 5 | agenow10 | 1630 (972, 2287) | <0.001 | 1540 (985, 2095) | <0.001 |
| F | 5 | agedx10 | 202 (-473, 878) | 0.557 | 138 (-428, 704) | 0.632 |
| F | 5 | simd_b | 317 (-191, 826) | 0.221 | 702 (270, 1135) | 0.001 |
| F | 5 | current_smoker | 1762 (996, 2528) | <0.001 | 1907 (1179, 2635) | <0.001 |
| F | 5 | total_chol | 55 (-214, 323) | 0.69 | 62 (-171, 295) | 0.6 |
| F | 5 | hdl | 157 (-602, 916) | 0.685 | 447 (-50, 945) | 0.078 |
| F | 5 | I(sbp/10) | 291 (96, 485) | 0.003 | 183 (18, 348) | 0.03 |
| F | 5 | ethnicasian | -1147 (-2506, 212) | 0.098 | -595 (-1565, 375) | 0.229 |
| F | 5 | ethnicother | 222 (-367, 810) | 0.461 | -656 (-1814, 502) | 0.261 |
| F | 5 | I(egfr/10) | -269 (-443, -94) | 0.003 | -337 (-491, -183) | <0.001 |
| F | 5 | I(hba1c/10) | 130 (-52, 312) | 0.161 | 219 (62, 376) | 0.006 |
| F | 5 | cv_disease | 1584 (787, 2380) | <0.001 | 1769 (1083, 2455) | <0.001 |
| F | 5 | bmi | 66 (25, 107) | 0.002 | 47 (9, 85) | 0.014 |
| Costs are from the complete-case analysis; Cost imputed and P value (imputed) represent pooled estimates based on Rubin’s rules across 10 imputed datasets; CI: confidence interval; SIMD: Scottish Index of Multiple Deprivation; M: Male; F: Female; agenow10: age at analysis (per 10 year increase); agedx10: age at T2DM diagnosis (per 10 year increase); total_chol: Total cholesterol; hdl: High-density lipoprotein cholesterol; I(sbp/10): Systolic blood pressure (per 10 mmHg increase); ethnicasian: Ethnicity – Asian; ethnicother: Ethnicity – Other; I(egfr/10): Estimated glomerular filtration rate (per 10 mL/min/1.73m² increase); I(hba1c/10): Glycated haemoglobin (HbA1c) (per 10 mmol/mol increase); bmi: Body mass index; cv_disease: History of cardiovascular disease; simd_b: Binary indicator for each SIMD quintile - coded as 0 for the lower decile (e.g., decile 1) and 1 for the higher decile (e.g., decile 2), as the original SIMD variable is in deciles. | | | | | | |

Table A7. Internal performance metrics for survival, complication, and cost models.

| Sex | SIMD | Gompertz model | Complication model (logistic regression) | Cost model (linear regression) | | |
| --- | --- | --- | --- | --- | --- | --- |
|  |  | C-stat | AUC* | R² | RMSE | MAE |
| 1 | 1 | 0.60997 | 0.673867 | 0.0173 | 16984 | 9367 |
| 1 | 2 | 0.613406 | 0.701813 | 0.0143 | 16697 | 8979 |
| 1 | 3 | 0.604711 | 0.696254 | 0.0134 | 18359 | 9306 |
| 1 | 4 | 0.599849 | 0.708369 | 0.0121 | 17121 | 9250 |
| 1 | 5 | 0.595808 | 0.710582 | 0.012 | 17562 | 9469 |
| 2 | 1 | 0.614387 | 0.690783 | 0.0186 | 16841 | 9297 |
| 2 | 2 | 0.616129 | 0.710484 | 0.0177 | 17029 | 9321 |
| 2 | 3 | 0.620509 | 0.734744 | 0.0159 | 16193 | 8954 |
| 2 | 4 | 0.613941 | 0.755038 | 0.0169 | 16746 | 9179 |
| 2 | 5 | 0.605382 | 0.748833 | 0.0231 | 16278 | 9189 |
| C-stat, concordance statistic; AUC, area under the receiver operating characteristic curve; R², coefficient of determination; RMSE, root mean squared error; MAE, mean absolute error; * AUC is reported for the stroke model as an example complication. | | | | | | |
